# Structural Variation and 3D Genome‐Driven DNA/RNA Methylation Divergence Contributing to Cotton Fiber Domestication

**DOI:** 10.1002/advs.202514381

**Published:** 2025-12-02

**Authors:** Lei Shao, Shangkun Jin, Haojie Jiang, Tianyu Pan, Zesheng Rui, Xiaowen Shi, Ting Zhao, Zhanfeng Si, Xueying Guan, Yan Hu, Tianzhen Zhang, Lei Fang

**Affiliations:** ^1^ Zhejiang Key Laboratory of Crop Germplasm Innovation and Utilization Key Laboratory of Speed Breeding in Plant Factory (Ministry of Agriculture and Rural Affairs) College of Agriculture and Biotechnology Zhejiang University Hangzhou 310058 China; ^2^ Hainan Institute of Zhejiang University Sanya 572025 China

**Keywords:** 3D chromatin, cotton fiber, DNA/RNA methylation, domestication, structural variation

## Abstract

Cotton domestication has driven remarkable improvements in fiber quality; however, the integrative roles of structural variation (SV), 3D genome architecture, and epigenetic regulation in this process remain poorly understood. Here, the genome assembly of wild (*yucatanense*, Yuc) and cultivated cotton (Xinluzao 61, XLZ61) is first reported. A total of 35 050 InDels and 165 inversions are identified, and 86.6% of these SVs are accumulated in differential topologically associating domain regions, indicating the important role of SV in 3D genome remodeling. Comparative analysis also yielded 199 359 differential DNA methylation regions and 2 092 787 differential RNA methylation loci, collectively affecting 44.7% of differentially expressed genes. Notably, SV‐associated methylation changes had stronger effects on gene expression than epigenetic modifications alone. Construction of an ultra‐dense genetic map comprising 13 602 620 SNP loci and QTL mapping further revealed SVs and 3D genome remodeling‐driven DNA/RNA methylation divergence to contribute to fiber length in domesticated cotton, demonstrating how SV reshapes trait variation. Collectively, these findings elucidated the crucial role of the “SVs‐3D genome remodeling‐epigenetic modifications‐gene expression” cascade regulatory network in cotton fiber domestication, offering both a theoretical foundation and genetic resources for molecular design breeding in fiber crops.

## Graphical abstract

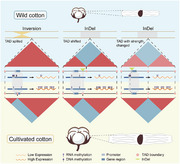

## 1 Introduction

Structural variations (SVs), including genome rearrangements such as inversions, which suppress recombination between locally adapted alleles, have long been hypothesized to play roles in species evolution, encompassing their origin, adaptation, and domestication.^[^ ^1^, ^2^, ^3^ ^]^ However, in early‐stage plant genome research, technological limitations and the lack of high‐quality reference genomes hindered the comprehensive study of structural variations; thus, the literature to date only focuses on QTL loci associated with SNPs and small InDels,^[^ ^4^, ^5^, ^6^ ^]^ with the role of large structural variations having been overlooked.^[^ ^7^ ^]^ This has resulted in a portion of missing heritability and an inability to adequately elucidate the genetic mechanisms underlying complex traits.^[^ ^8^, ^9^, ^10^ ^]^ With advances in sequencing technologies, pan‐genome studies have begun employing multiple high‐quality assemblies and can now delineate genomic variation inaccessible to single‐genome analyses. Such work has revealed that SVs in coding or regulatory regions can modulate gene expression, thereby altering agronomic traits.^[^ ^7^, ^9^, ^11^, ^12^, ^13^, ^14^, ^15^, ^16^ ^]^ Usually, SVs act as cis‐regulatory switches through repositioning promoters and enhancers^[^ ^12^, ^17^ ^]^ or rewiring 3D genome architecture^[^ ^18^, ^19^, ^20^, ^21^ ^]^ to fine‐tune spatiotemporal expression of genes. Concurrently, epigenetic mechanisms such as DNA methylation, histone modifications, and RNA methylation regulate phenotypic plasticity through dynamic control of gene expression without alteration of DNA sequence.^[^ ^22^, ^23^, ^24^, ^25^, ^26^ ^]^ Recent advances in methylation detection technologies have provided unprecedented tools for elucidating underlying mechanisms.^[^ ^27^, ^28^, ^29^ ^]^

Cotton (*Gossypium* spp.), one of the earliest domesticated crops in agricultural history, has been a cornerstone of global textile production. Among extant species, allotetraploid upland cotton (*Gossypium hirsutum* L., AD genome) dominates commercial cultivation, accounting for over 90% of the world's renewable textile fiber.^[^ ^30^ ^]^ This species originated from the domestication of its wild progenitor, *G. hirsutum* race yucatanense (Yuc), ≈5000 years ago.^[^ ^31^ ^]^ The domestication syndrome in cotton encompasses three major phenotypic transitions: 1) photoperiod response transition from photoperiod‐sensitive to photoperiod‐insensitive, 2) morphological shifts from perennial tall shrubs/small trees to annual cultivated shrubs, and 3) fiber trait optimization involving the elongation and refinement of initially short, coarse fibers. In the course of elucidating the genetic basis of phenotypic variation in cotton, extensive SNP‐based studies have identified critical domestication loci related to fiber quality, yield, and seed germination;^[^ ^6^, ^32^, ^33^ ^]^ in addition, comparative genomic analysis has revealed critical roles for SVs in cotton photoperiod response and fiber domestication.^[^ ^34^, ^35^ ^]^ Beyond such sequence variations, epigenetic modifications also made essential contributions to cotton domestication. For example, DNA methylation reprogramming modulates photoperiod sensitivity, potentially enabling adaptation to diverse agroecological environments during domestication,^[^ ^36^ ^]^ and N^6^‐methyladenosine (m^6^A) has been demonstrated to modulate fiber elongation and photoperiod sensitivity by regulating mRNA stability of key genes.^[^ ^37^, ^38^ ^]^ However, while such prior studies have explored the independent roles of genetic and epigenetic factors in cotton domestication, the molecular mechanisms by which SVs orchestrate epigenetic reprogramming to enhance fiber quality remain to be delineated.

Here, recognizing the critical need for systematic multi‐omics integration in illuminating cotton domestication, we selected and assembled the wild species Yuc and the elite cultivar XLZ61 to establish a comparative framework. Multi‐omics analyses, including 3D genome architecture, transcriptome dynamics, and epigenome profiling, were conducted on fiber samples collected at 10 and 20 days post‐anthesis (DPA), corresponding to the key developmental phases of fiber elongation and secondary cell wall (SCW) deposition. We further constructed an F_2_ population from Yuc and XLZ61 and performed bulk segregant analysis (BSA) sequencing and genetic linkage map‐based QTL mapping, leading to the identification of a novel domestication QTL locus (*qFL/A07*) and the pinpointing of key candidate genes. All told, this study sheds light on the role of SV‐mediated 3D genome remodeling and epigenetic modifications (DNA and RNA methylation) in facilitating changes that occurred during cotton fiber domestication.

## 2 Results

### 2.1 The Domesticated Characteristics of Cotton Fiber

**Figure 1:**
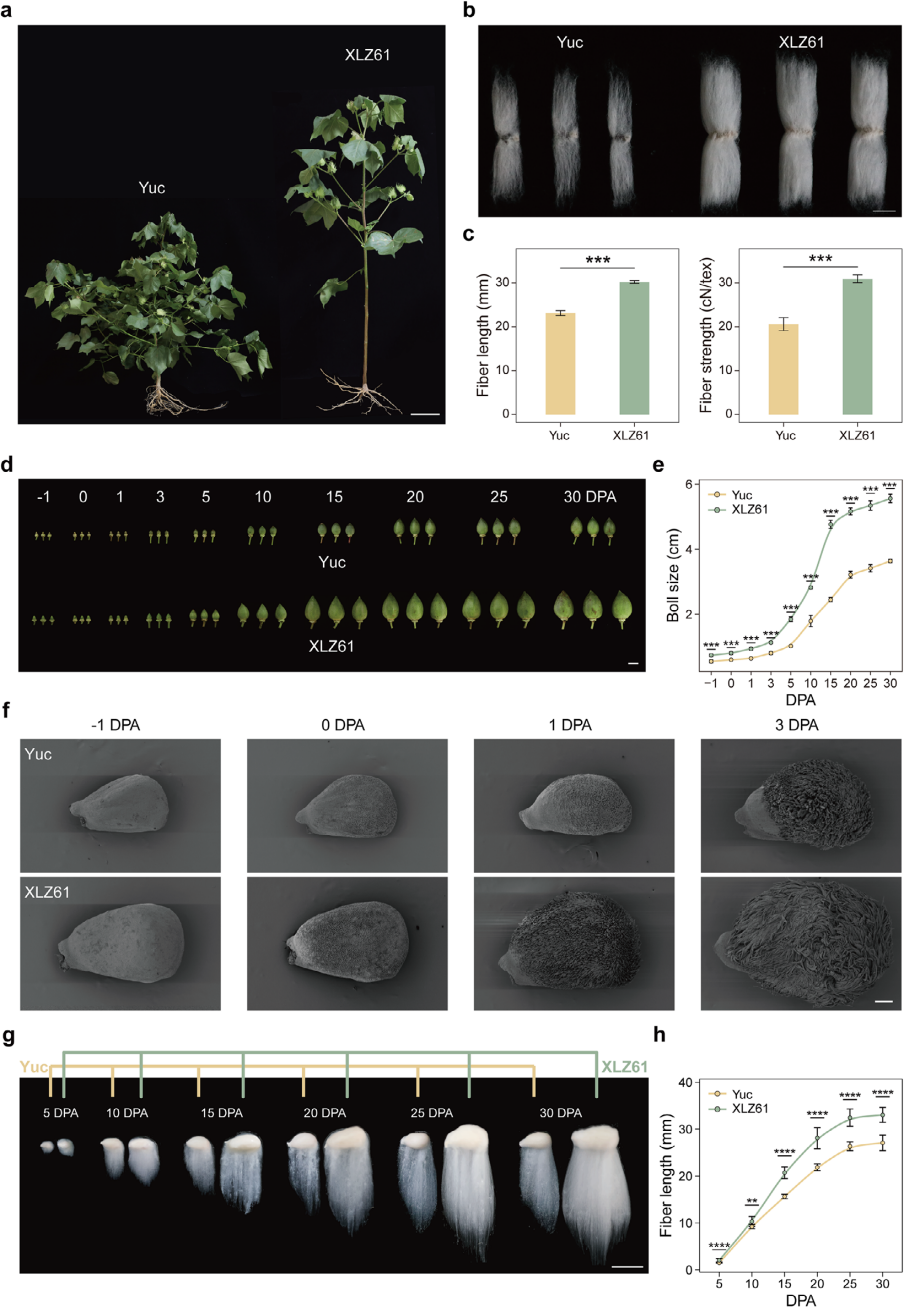
The domesticated characteristics of cotton. a) Plant architecture phenotype in Yuc and XLZ61 (The photo was taken in Sanya, Hainan province). Yuc exhibits extreme sensitivity to chlormequat, resulting in significantly suppressed plant height. b) Fiber length phenotypes in Yuc and XLZ61. c) Box plot of fiber length and fiber strength in Yuc and XLZ61. d) Boll phenotypes in Yuc and XLZ61. Scale bar, 10 mm. e) Boll size patterns for Yuc and XLZ61. Each value is the mean ± s.d. of fiber length for at least ten bolls from three individual plants at the given time point. ^***^ indicated *P* value <0.001. f) Scanning electron microscopy (SEM) of Yuc and XLZ61 ovules at −1, 0, 1, 3 DPA. Scale bar, 200 mm. g) Fiber‐bearing seed phenotypes in Yuc and XLZ61. Numbers above the graphs are DPA. Scale bar, 10 mm. h) Fiber elongation patterns for Yuc and XLZ61. Each value is the mean ± s.d. of fiber length for at least ten seeds from three individual plants at the given time point. ^****^ indicated *P* value <0.0001, ^**^ indicated *P* value <0.01.

Yuc and XLZ61 cotton plants exhibited distinct phenotypic differences, such as in response to chlormequat chloride sensitivity (resulting in plant height difference) (**Figure** **1**a), fiber quality (Figure 1b,c), and boll size (Figure 1d,e). We visualized fibers at the initiation stage (−1, 0, 1, 3 DPA) by scanning electron microscopy and found the number of protruding cells in XLZ61 to be more than in Yuc (Figure 1f). Measurements of dynamic changes in the elongating fibers of XLZ61 and Yuc plants from 5 to 30 DPA (Figure 1g) revealed both to undergo a nearly linear increase in fiber length over 5–25 DPA, but the rate of growth is higher in XLZ61 than in Yuc (Figure 1h), leading to the development of longer fibers in XLZ61 (Figure 1h). These findings indicated that fiber developmental processes were greatly altered during domestication.

We next collected samples from both plants for RNA sequencing: cotton leaves, ovules at −1, 0, 1, 3, and 5 DPA, and fiber samples at 5, 10, 15, 20, 25, and 30 DPA (Table S1, Supporting Information). It was found that over the course of fiber development, the total number of expressed genes decreased (Figure S1, Supporting Information). In total, we identified 20 344 differentially expressed genes (DEGs), including 9247 at −1 DPA, 6056 at 0 DPA, 3611 at 1 DPA, 6633 at 3 DPA, 5799 at 5 DPA, 3971 at 10 DPA, 2893 at 15 DPA, 3556 at 20 DPA, 3502 at 25 DPA, and 2953 at 30 DPA (Figure S2a, Supporting Information). Gene Ontology (GO) enrichment analysis showed these DEG groups to be enriched in biological processes related to fiber development (Figure S2b, Supporting Information). For example, the fatty acid biosynthetic process was enriched in the elongation stage (15, 20 DPA). Among genes associated with this process, *GH_A03G1485* showed higher expression in XLZ61 than in Yuc (Figure S2c, Supporting Information). Analysis of its expression at the population level revealed *GH_A01G1813* to be more highly expressed in cultivated species (*n* = 207) than in wild/semi‐wild species (*n* = 24) (Figure S2d, Supporting Information). Other notable terms included transporter activity, secondary cell wall biogenesis, and cellulose biosynthetic process, which were enriched in the SCW thickening stage (20, 25, 30 DPA). *GH_A01G1813*, which was annotated with transporter activity process, showed higher expression in cultivated species than in wild species (Figure S2e,f, Supporting Information). These results suggest that genes differentially expressed in the ovule and fiber between Yuc and XLZ61 are likely to have made functional contributions to fiber improvement during cotton domestication.

### 2.2 Effects of Structural Variation on Topologically Associating Domains During Domestication

**Figure 2:**
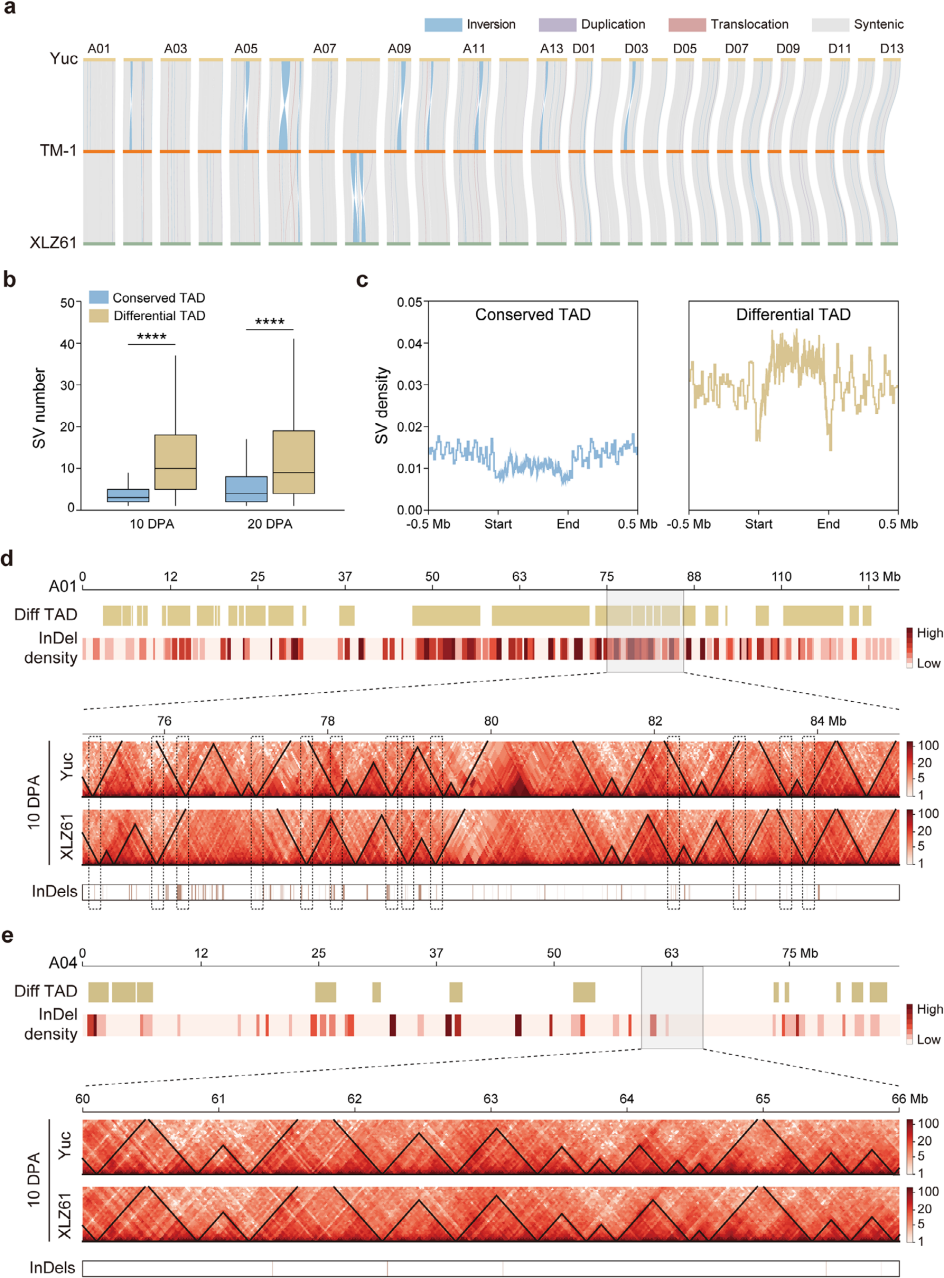
Effects of structural variation on topologically associating domains during domestication. a) Genome alignment among Yuc, TM‐1, and XLZ61. Vertical lines represent syntenic (grey), inverted (blue), translocated (pink), and duplicated (purple) regions, respectively. b) Box plot of SV number in conserved and differential TADs. The center line denotes the median; box limits indicate the upper and lower quartiles; whiskers mark the 1.5X interquartile range, and points show outliers; n indicates the number of individuals. The significance of SV number differences was determined by a two‐tailed *t*‐test. ^****^ indicated *P* value <0.0001. c) Plots of SV density around conserved and differential TADs. d) Representative hot spot of differential TADs associated with high SV density. e) Representative cold spot of differential TADs with few SVs.

We assembled the Yuc and XLZ61 genomes by integrating Illumina short‐read, PacBio high fidelity (HiFi) read, and high‐throughput chromosome conformation capture (Hi‐C) sequencing (Tables S2 and S3, Supporting Information). We produced 92.9 Gb HiFi reads for Yuc (≈40× genome equivalents) and 98.1 Gb HiFi reads for XLZ61 (≈43× genome equivalents) using the PacBio Sequel II platform (Table S2, Supporting Information). These HiFi reads were first used to generate an initial Yuc genome assembly (contig N50 = 98.3 Mb) and an XLZ61 genome assembly (contig N50 = 87.7 Mb). The contigs were then anchored and oriented into a final assembly using chromatin interaction maps generated from Hi‐C data (≈182.2× for Yuc and ≈179.4× for XLZ61). The final Yuc assembly captured 2387.7 Mb with a scaffold N50 of 108.4 MB, while the XLZ61 assembly captured 2333.4 Mb with a scaffold N50 of 108.5 Mb. The uniform genome coverage of HiFi reads (Figure S3, Supporting Information) and the Hi‐C heat maps (Figure S4, Supporting Information) support the chromosome‐scale structure. The base accuracy of the two assemblies was estimated to be over 99.99% (quality value score 48.5 for Yuc and 47.6 for XLZ61) based on mapped K‐mers using Illumina data^[^ ^39^ ^]^ (Figure S5, Supporting Information). We compared these two assemblies against the TM‐1 assembly^[^ ^30^ ^]^ using the minimap2 tool^[^ ^40^ ^]^ and identified SVs using the SyRI tool.^[^ ^41^ ^]^ We next merged three types of SVs (insertion, deletion, and inversion) into a set of nonredundant SVs using SURVIVOR.^[^ ^42^ ^]^ In total, 16 832 insertions, 18 218 deletions, and 165 inversions were identified between Yuc and XLZ61 (**Figure** **2**a; Figure S6, Supporting Informationa). Of these SVs, 1004 were located in exons (2.87%), 1686 in introns (4.83%), 1448 upstream of a gene (4.15%), 1375 downstream (3.94%), and the remaining 29 300 in intergenic regions (83.87%) (Figure S6b, Supporting Information).

To investigate chromatin topology, we produced Hi‐C data of fiber tissue at 10 and 20 DPA for both Yuc and XLZ61 (Table S3, Supporting Information). This yielded total uniquely aligned contact read counts of 36 030 4103 and 470 234 991 for Yuc and 289 743 618 and 334 859 084 for XLZ61 at the respective developmental stages. When examined at various resolutions, Yuc and XLZ61 showed different higher‐order chromatin structures (Figure S7, Supporting Information). We next categorized the chromatin at each developmental stage into A and B compartments using the 40 Kb Hi‐C matrix. As expected, A compartments were mainly located in chromosome arms with higher gene expression and lower DNA methylation levels (Figure S8a, Supporting Information), while B compartments were located in centromeric and pericentromeric regions, showing lower gene expression and higher DNA methylation levels (Figure S8b, Supporting Information). Using the 20 Kb resolution matrices, we identified 4316 and 3654 TADs in Yuc and 4414 and 5622 TADs in XLZ61 at 10 and 20 DPA, respectively. The TAD boundaries showed lower DNA methylation and higher gene expression (Figure S9a,b, Supporting Information).

Little is yet known about the effects of SVs on higher‐order chromatin structures during domestication. We investigated the dynamic switching of A/B compartment status between wild and cultivated cotton. Overall, most chromatin compartments, including 35.7% of genome length in A compartments and 59.5% of genome length in B compartments, remained stable. Only 4.8% of the genome length showed A/B compartment shifts (Figure S10a, Supporting Information). Compared to genes in conserved A compartments, genes with A to B shift from wild species to cultivar showed a higher ratio of down‐regulated genes and a higher level of methylation (Figure S10b, Supporting Information). Opposite results relative to conserved B compartments were observed for genes with B to A shift (Figure S10b, Supporting Information). Comparative analysis identified 1623 differential TADs and 2693 conserved TADs at 10 DPA, along with 1512 differential TADs and 2142 conserved TADs at 20 DPA. The fold change in gene expression within differential TADs is significantly greater than that within conserved TADs, demonstrating that alterations in TAD structure impact gene expression levels (Figure S11, Supporting Information). SVs were found to be significantly enriched in differential TADs (Figure 2b,c), and were more likely to occur within the TAD interior than in TAD boundaries (Figure 2c). Differential TAD‐like structures were unevenly distributed across the genome (Figure S12, Supporting Information); for example, chromosome A01 harbored a 75.9 Mb region of differential TAD‐like structures that accounted for 64.3% of the total chromosome length. To more clearly visualize the relationship between SVs and differential TAD‐like structures, the 75–85 Mb interval was enlarged. Within this interval, 18 differential TAD boundaries were identified, 13 of which coincided with SVs (Figure 2d). As a contrasting example, on chromosome A04, differential TAD‐like structures covered only 20.3% of the chromosome length. Furthermore, within the 60–66 Mb interval on A04, no significant changes in TAD‐like structures occurred, and only four SVs were present (Figure 2e). We next performed a genome‐wide analysis to quantify the overlap between TAD boundaries and SVs. We divided the genome into 205 non‐overlapping 10‐Mb windows and calculated the proportion of TAD boundaries overlapping with SVs for each window. The average proportion of SV‐associated TAD boundaries across all windows was 55% (Figure S13, Supporting Information). These results indicate that SVs played critical roles in driving alterations in TAD‐like domains during cotton fiber domestication.

### 2.3 DNA Methylation Footprints During Cotton Domestication

To uncover DNA methylation changes during cotton domestication, we generated single‐base‐resolution methylomes for Yuc and XLZ61 leaf, 10 DPA fiber, and 20 DPA fiber using whole‐genome bisulfite sequencing, with three biological replicates. A total of 1.39 Tb clean data with an average coverage depth of 33.6× was produced (Table S4, Supporting Information). These clean reads were aligned to the TM‐1 genome,^[^ ^30^ ^]^ and successfully identified three types of methylation modification (CG, CHG, CHH). The three replicates of each sample consistently showed a high Pearson correlation coefficient (*r*), implying reproducible coverage (Figure S14, Supporting Information). Of the three cytosine contexts, CG methylation predominated in all samples (Table S5, Supporting Information). From the chromosome‐scale viewpoint, methylation sites were enriched in heterochromatic regions (Figure S15, Supporting Information). With respect to genomic regions, DNA methylation was distributed at distinct regions, including protein‐coding genes, promoters, and transposable elements (TEs). Notably, promoter regions displayed higher DNA methylation levels than protein‐coding regions, and TE regions displayed higher DNA methylation levels than non‐TE regions (Figure S16, Supporting Information). This observed methylation pattern is consistent with the well‐conserved epigenetic landscape reported across numerous plant species, including Arabidopsis,^[^ ^43^ ^]^ soybean,^[^ ^44^ ^]^ rice,^[^ ^45^ ^]^ and maize.^[^ ^46^ ^]^ This conservation underscores the fundamental role of these methylation patterns in maintaining genome stability and regulating gene expression in plants.

To investigate differences in DNA methylation patterns associated with cotton domestication, we compared XLZ61 to Yuc in terms of DNA methylation sites and levels. No significant change was observed in the number of CG, CHG, and CHH methylation sites. To further examine patterns of variation in DNA methylation, we divided the genome into non‐overlapping 1000 bp regions and identified differentially methylated regions (DMRs). In total, 84563‐92983 CG‐DMRs, 76254‐101494 CHG‐DMRs, and 16863‐159844 CHH‐DMRs were identified for the various stages (Table S6, Supporting Information). From the chromosome‐scale perspective, CG‐DMRs and CHH‐DMRs were mainly concentrated at chromosome ends with protein‐coding genes, while CHG‐DMRs exhibited a uniform distribution across the chromosomes (Figure S17, Supporting Information). With regard to genomic regions, CG‐DMRs tended to occur in gene bodies and TE regions, whereas CHG‐DMRs and CHH‐DMRs tended to occur in TE and intergenic regions (Figure S18, Supporting Information).

To examine whether DNA methylation changes influenced gene expression during cotton fiber domestication, we identified 19938‐36823 DMR‐associated proximal genes (DPGs), defined as having DMRs located within 2 Kb upstream of the transcription start site (Table S7, Supporting Information). This revealed DNA methylation changes to have significantly induced expression changes (Figure S19, Supporting Information). Among these DPGs, a total of 3917 genes, by combining those identified across different periods, exhibited differential expression. For example, *GH_D04G1517*, encoding adenine nucleotide alpha hydrolases‐like superfamily protein, showed higher methylation levels across its promoter and gene body region in XLZ61 at 10 and 20 DPA (Figure S20a, Supporting Information), resulting in its non‐expression (Figure S20b, Supporting Information). Further analysis of its expression at the population level revealed *GH_D04G1517* to be hardly expressed in cultivated species (*n* = 193), with notable expression only in wild/semi‐wild species (*n* = 24) (Figure S20c, Supporting Information). This indicates that *GH_D04G1517* is a gene targeted by selection during domestication with differential expression affected by methylation. We next performed GO enrichment analysis on the DMR‐associated DEGs. The results revealed enrichment for several biological pathways, including cell redox homeostasis, microtubule‐based processes, and structural constituents of the cytoskeleton, all of which are implicated in fiber development (Figure S21, Supporting Information).

### 2.4 RNA Methylation Footprints During Cotton Domestication

**Figure 3:**
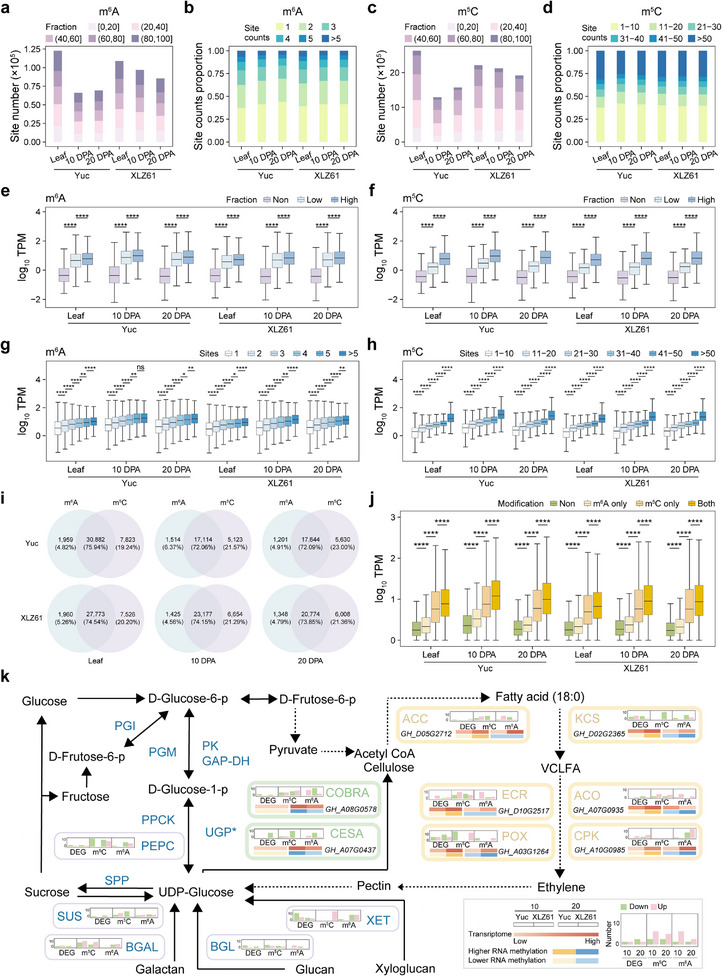
RNA methylation footprints during cotton domestication. a) The number of m^6^A‐modified sites. Each site was classified into different categories on the basis of its fraction. b) Ratio of transcripts with a different number of m^6^A‐modified sites. Each color corresponds to sites number in each transcript. c) The number of m^5^C‐modified sites. Each site was classified into different categories on the basis of its fraction. d) The ratio of transcripts with a different number of m^5^C‐modified sites. Each color corresponds to sites number in each transcript. e) Relationship of m^6^A modification level with transcript expression level. High, Low, and Non indicated the transcripts with the maximum fraction ranging from 0.5 to 1.0, 0.0 to 0.5, and without m^6^A modification, respectively. f) Relationship of m^5^C modification level with transcript expression level. g) Relationship of m^6^A modification sites with transcript expression level. Site represented the transcripts with RNA modification, number represented the number of RNA modification sites in each transcript. h) Relationship of m^5^C modification sites with transcript expression level. i) Venn plot of transcripts with m^6^A and m^5^C in different tissues. j) The expression level of transcripts with different modifications. Both indicate the transcripts modified by both m^6^A and m^5^C; m^6^A only indicates the transcripts modified by m^6^A only; m^5^C only indicates the transcripts modified by m^5^C only; no indicates the transcripts without m^6^A and m^5^C modifications. k) Comparative analysis of RNA modification and expression levels of gene families within the sugar metabolism pathway. The significance was determined by a t‐test. ^****^ indicated the *p* < 0.0001, ^**^ indicated the *p* < 0.01 and ^*^ indicated the *p* < 0.05.

To obtain a comprehensive view of the cotton transcriptome and its dynamics in relation to domestication, we applied Nanopore direct RNA sequencing (DRS) to Yuc and XLZ61 leaf, 10 DPA fiber, and 20 DPA fiber samples. A total of 85.7 Gb data were generated (Table S8, Supporting Information). The high Pearson correlation coefficient (*r*) between the two replicates of each sample (Figure S22, Supporting Information) implied reproducible coverage. As a new technology, DRS reads enable the identification of m^6^A and 5‐methylcytosine (m^5^C) modifications, which are not only the most prevalent post‐transcriptional modifications but also essential for regulating gene expression.^[^ ^23^, ^37^, ^38^ ^]^ In this study, we successfully identified m^6^A and m^5^C across six distinct tissues using biological replicates (*n* = 2). The methylation levels between replicate pairs showed correlation coefficients ≥0.8, demonstrating high consistency of DRS in detecting RNA modifications (Figure S23, Supporting Information). At the overall genomic level, a total of 161651 (Yuc) and 170929 (XLZ61) m^6^A‐modified sites were identified within 62804 (Yuc) and 66595 (XLZ61) transcripts; at the tissue level, the number of sites ranged from 65715 (Yuc 10 DPA) to 120973 (Yuc leaf) (**Figure** **3**a; Table S9, Supporting Information). Most transcripts had less than three m^6^A sites (Figure 3b). The fraction of transcripts with more than five modified sites did not display a different variation, but the maximum fraction in these transcripts was significantly higher than that in other modified transcripts (Figure S24, Supporting Information). For m^5^C modification, at the genome level, a total of 3 438 320 (Yuc) and 3 628 677 (XLZ61) m^5^C‐modified sites were identified within 73 617 (Yuc) and 78281 (XLZ61) transcripts, with the number of sites at the tissue level ranging from 1 245 698 (Yuc 10 DPA) to 2 548 813 (Yuc leaf) (Figure 3c; Table S9, Supporting Information). Transcripts with fewer than ten or more than 50 m^5^C sites were the most abundant (Figure 3d), and as the number of m^5^C sites increased, so did the maximum level of methylation modification (Figure S25, Supporting Information).

To understand the function of m^6^A and m^5^C, we analyzed the correlation of modification presence with transcript expression level. The results demonstrated m^6^A‐ or m^5^C‐modified transcripts to have significantly higher expression levels than transcripts with no modification, and transcripts with higher fractions of modification sites also had higher expression (Figure 3e,f). Furthermore, transcripts with more m^6^A or m^5^C sites tended to have higher expression (Figure 3g,h). The number of co‐modified transcripts (with both m^6^A and m^5^C modifications) varied significantly across tissues and accessions, ranging from 16953 in XLZ61 fibers at 10 DPA to 30343 in XLZ61 leaves (Figure 3i). Comparative analysis revealed that 91.87–94.21% of m^6^A‐modified transcripts were simultaneously modified by m^5^C, while 75.81–79.79% of m^5^C‐modified transcripts also carried m^6^A modifications (Figure S26, Supporting Information). To investigate whether m^6^A and m^5^C modifications act synergistically to enhance expression, the expression levels of transcripts carrying both modifications were compared to those carrying only one or no modification. This showed transcripts with dual modifications to exhibit significantly higher expression than those with only m^5^C or only m^6^A modifications (Figure 3j).

To investigate differences in RNA methylation patterns associated with cotton domestication, we further identified differentially methylated loci. In total, 871 183–1 305 084 differentially methylated m^5^C sites were identified, accounting for 38.2–42.2% of total sites, along with 47170 to 69874 differentially methylated m^6^A sites, representing 44.5–46.1% of total sites (Table S10, Supporting Information). Differentially m^5^C‐methylated sites were located on 11 427–23 042 differentially expressed transcripts (DETs), while the differentially m^6^A‐methylated sites were located on 6873–14 687 DETs. Transcripts harboring differential RNA methylation showed significant enrichment in sugar‐metabolism‐related functions, which are closely associated with fiber development (Figure S27, Supporting Information). Comparative analysis of RNA modification levels and expression levels of gene families (752 genes) within the sugar metabolism pathway^[^ ^47^ ^]^ between Yuc and XLZ61 revealed 418 genes (55.6% of the total analyzed) to exhibit significant differences in RNA modification, of which 104 genes showed significant changes in gene expression. For instance, at the 20 DPA stage, key genes related to cellulose synthase (CesA and COBAR) showed significantly reduced RNA modification in XLZ61, leading to significantly downregulated expression (Figure 3k). Conversely, key genes in the ethylene biosynthesis pathway (such as ACC, ECR, KCS, POX) exhibited significantly increased RNA modification in XLZ61, resulting in significantly upregulated expression (Figure 3k). These results indicate that RNA modification may have played a critical role in regulating the sugar metabolism pathway during cotton fiber domestication.

### 2.5 The Molecular Mechanisms by which SVs Affect Gene Expression

**Figure 4:**
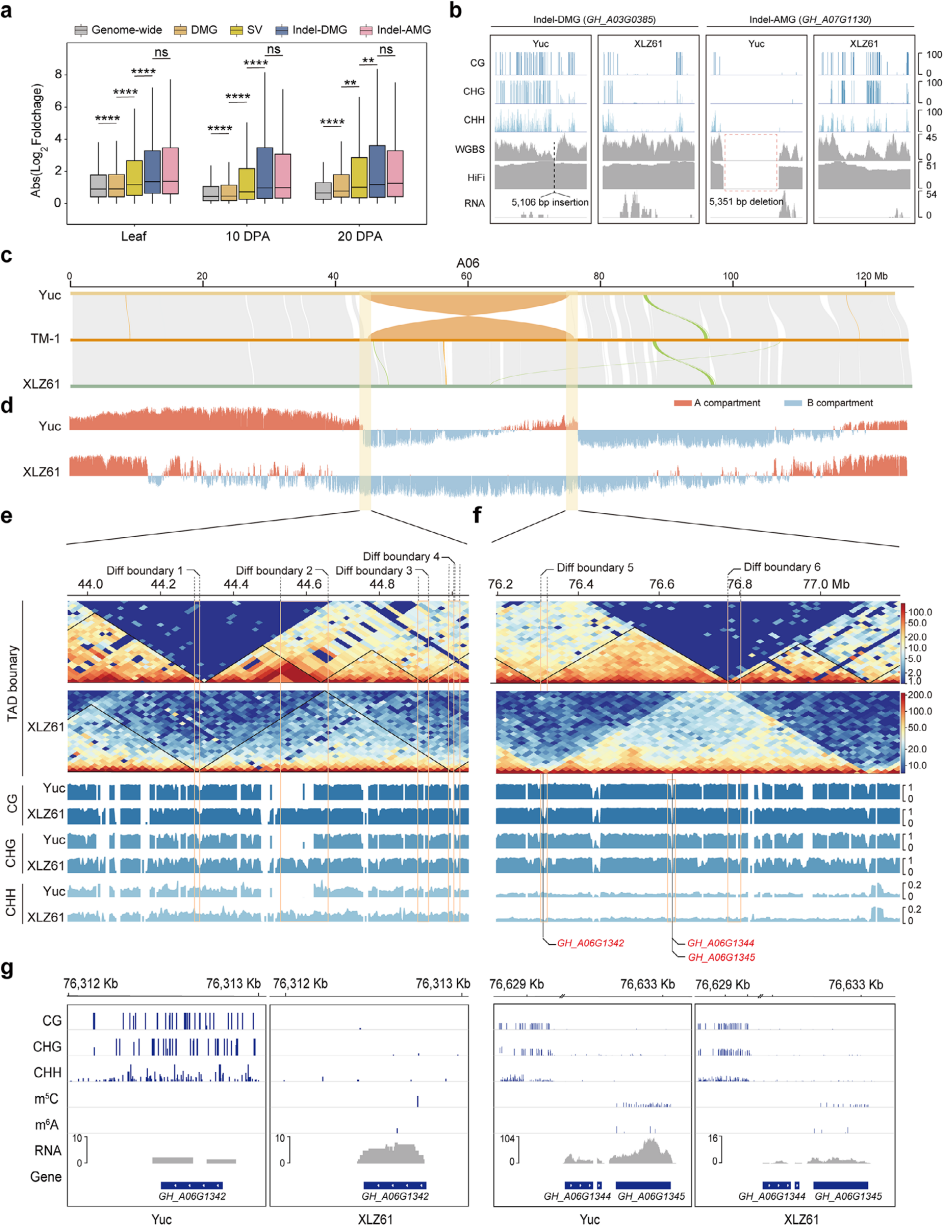
SVs influence gene expression through DNA/RNA methylation effects. a) Box plot of gene expression changes (absolute values) across the genome‐wide and in SV‐genes, DMGs, InDel‐DMGs, and InDel‐AMGs in leaf tissue and 10 and 20 DPA fiber. The significance was determined by a t‐test. ^****^ indicated the *p* < 0.0001 and ^**^ indicated the *p* < 0.01. b) Example of gene expression modulation via InDel‐associated DMR and InDel‐mediated AMR. c) Genome alignment among Yuc, TM‐1, and XLZ61 in A06 chromosome. A large inversion was identified. d) A/B compartment status across chromosome A06. e) Plot of the differential TAD region and differential DNA methylation in the left border of the inversion. f) Plot of the differential TAD region and differential DNA methylation in the right border of the inversion. g) Plot of the DNA and RNA methylation of DEGs in differential TAD regions.

While SVs, alterations in TADs, and DNA methylation are all known to influence gene expression, the precise mechanisms through which these factors coordinately regulate transcriptional output remain poorly understood. First, we defined a gene as an “InDel‐DMG” when it concurrently harbors both an InDel and a DMR. A total of 2415‐3236 InDel‐DMGs were identified (Table S11, Supporting Information; **Figure** **4**b). We found InDel‐DMGs to have a higher magnitude of differential expression than genes with only DMR or InDels (Figure 4a). Furthermore, this study defined a novel category of acquired methylation region (AMR) directly introduced by InDels (InDel‐AMR). Distinct from real epiallelic variation that occurs in identical DNA sequences (Figure 4b), this type of methylation divergence primarily stems from epigenetic modifications carried by the SV fragments themselves, representing a sequence‐dependent methylation variation (Figure 4b). A total of 1140–1377 InDel‐AMR‐associated genes (InDel‐AMGs) were identified (Table S11, Supporting Information; Figure 4b). InDel‐AMGs also contribute to enhanced differential gene expression, with a magnitude comparable to that of InDel‐DMGs (Figure 4a). These results suggested that InDel could exert a greater effect on gene expression by driving methylation divergence. We then proceeded to examine the effects of inversions on DNA methylation patterns. Within the inversion regions, a total of 801 genes were expressed in at least one material, among which 78 genes exhibited differential expression, accounting for 9.73% of the total. This proportion is higher than the percentage of DEGs in the entire genome (8.70%). Among these 78 DEGs, 59 showed differential methylation modifications. Subsequently, we investigated the distribution of DMRs within the inversion and found a higher density of DMRs at the boundaries of differential TADs (Figure S28, Supporting Information). Therefore, we speculate that inversions may also affect a TAD boundary, which in turn alters methylation levels. For example, a large inversion (≈32 Mb) between Yuc and XLZ61 was identified on A06 (Figure 4c), consistent with previously reported inversions in HPF17 (semi‐wild cotton) and TM‐1.^[^ ^34^ ^]^ We found the breakpoints of this inversion to alter the A/B compartment (Figure 4d) and TAD status (Figure 4e,f). At the left border (± 500 Kb), four TADs with differences between Yuc and XLZ61 at 10 DPA were identified that led to four distinct boundaries, which in turn altered DNA methylation levels between Yuc and XLZ61 (Figure 4e). At the right border (± 500 Kb), two differential TADs and two distinct boundaries were similarly found to alter DNA methylation (Figure 4f). Further investigation revealed differential TADs accompanied by differential methylation modifications to harbor five genes. Among these, *GH_A06G1329* and *GH_A06G1331* exhibited no detectable expression during fiber development stages, whereas *GH_A06G1342*, *GH_A06G1344*, and *GH_A06G1345* showed significant differential expression at 10 DPA (Figure 4g). Notably, in addition to differences in DNA methylation modifications, *GH_A06G1342* and *GH_A06G1345* displayed concurrent differences in RNA methylation modifications (Figure 4g). Moreover, we found that this inversion already exists in some semi‐wild accessions, and the expression patterns of the three genes in the accessions with the inversion are consistent with those in the XLZ61 (Figure S29, Supporting Information). These results indicate that during cotton domestication, SVs can modulate gene expression by altering DNA or RNA methylation levels through 3D genome remodeling.

### 2.6 SVs and 3D Genome Variations Orchestrate DNA/RNA Methylation to Potentiate Fiber Length Domestication

**Figure 5:**
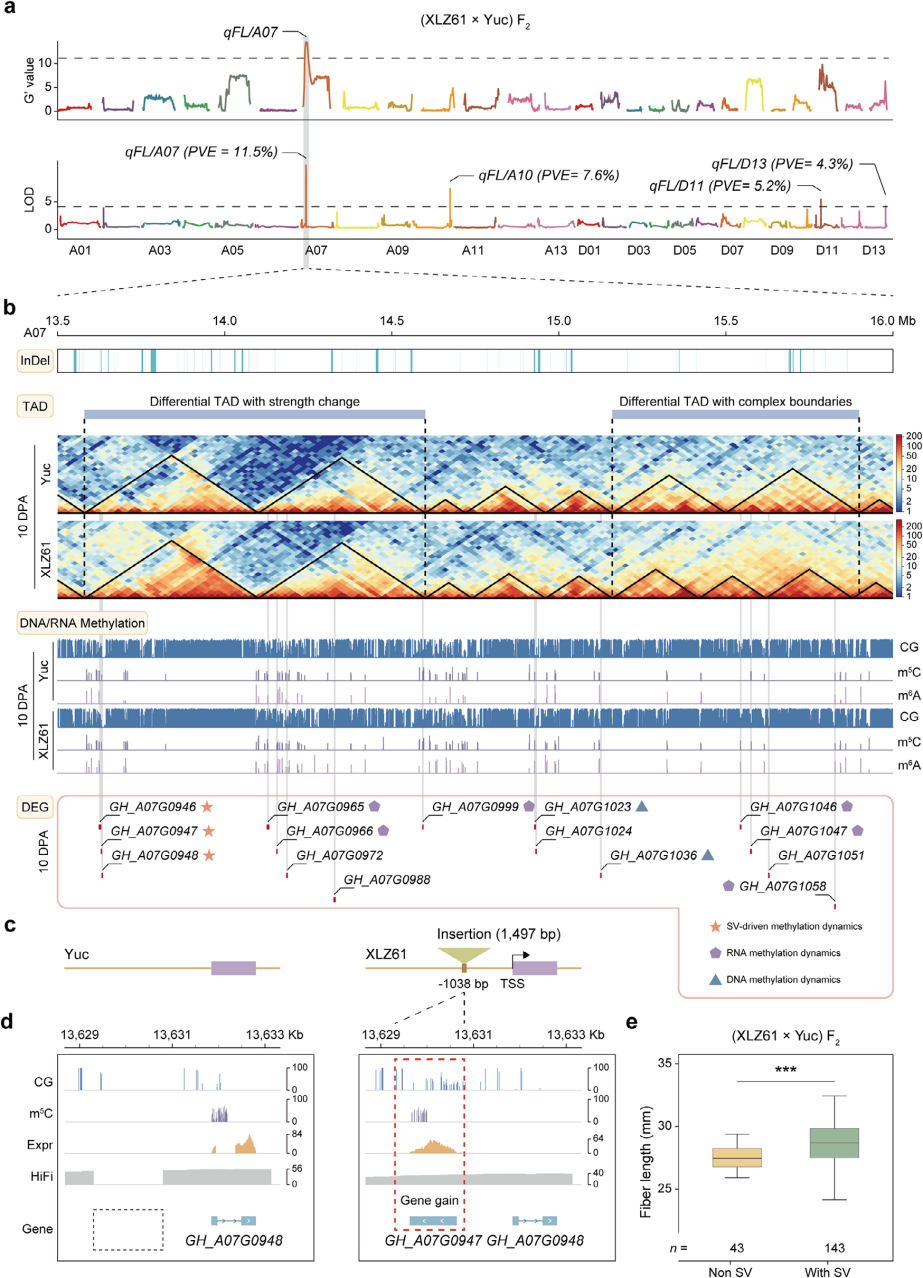
SV and 3D genome variations coordinate DNA/RNA methylation to enhance fiber domestication. a) Identification of QTLs related to fiber domestications by BSA and genetic‐map‐based QTL mapping. *qFL/A07*, a novel QTL, was co‐detected by two independent methods. b) Plot of the InDel distribution, the TAD structure of Yuc and XLZ61, the DNA and RNA methylation level of Yuc and XLZ61, and the DEGs in the *qFL/A07* region. c) An insertion with a 1,497 bp size occurred in the promoter of *GH_A07G0948* in XLZ61. d) The impact of the acquired methylation region mediated by a 1,497 bp insertion on gene expression. e) Box plot of fiber length in wild‐type (without SV) and cultivar‐type (with SV) individuals from a F_2_ population. The significance was determined by a t‐test. ^***^ indicated the *p* < 0.001.

To illustrate how complex trait variation is influenced by the SV‐gene expression associations established above, we constructed an F_2_ mapping population comprising 586 individuals from crosses between XLZ61 and Yuc and investigated their fiber traits: fiber length (FL), fiber strength (FS), fiber elongation (FE), fiber micronaire (FM), and fiber uniformity (FU). First, the 30 individuals with the worst fiber quality and the 30 individuals with the best fiber quality were selected for bulked segregant analysis sequencing (BSA‐seq) (Table S12, Supporting Information). This revealed a QTL in chromosome A07 (*qFL/A07*) to be responsible for fiber length (**Figure** **5**a), and four QTLs in A07, D09, D11 (*qFS/A07*, *qFS/D09, qFS/D10*, and *qFS/D11*) to be responsible for fiber strength (Figure S30 and Table S13, Supporting Information). Second, 300 randomly‐selected individuals were sequenced at an average depth of 15× reads (Table S14, Supporting Information). The clean reads were aligned to the TM‐1 genome, and after filtering, a total of 13 602 620 high‐quality SNPs were obtained. These SNPs were assigned into 4733 bin markers to construct a high‐density genetic map covering 4192.7 cM (Figure S31 and Table S15, Supporting Information). The genetic and physical maps showed good collinearity (Figure S32, Supporting Information). QTL mapping was then conducted for fiber quality‐related traits, which yielded ten major QTLs with 3.78–22.93% of phenotypic variance explained (Table S16 and Figure S33, Supporting Information). By integrating two complementary approaches, we identified a total of 14 fiber‐quality‐related QTLs. Among these, *qFL/A10*, *qFL/D11*, and *qFL/D13* were consistent with previously reported results,^[^ ^48^ ^]^ confirming the reliability of the mapping in this population. Notably, a further novel locus on chromosome A07 was identified, associated with fiber length (Figure 5a).

As regards the *qFL/A07* locus (13.62–15.98 Mb) in particular, 76 SVs, four differential TADs, 151 DMRs, and 1432 DMLs (Figure 5b) were identified with a total of 15 genes showing differential expression at 10 DPA (Figure 5b). Among these, an SV, 1,497 bp insertion, mediated acquired methylation region was identified and occurred in the promoter of *GH_A07G0948* in XLZ61 (Figure 5c,d). This SV was validated in HiFi reads mapped to the TM‐1 genome assembly (Figure 5d), and the insertion fragment was annotated as a presence‐absence variation (PAV) gene, designated *GH_A07G0947*, which is expressed in the XLZ61 accession. Intriguingly, this PAV gene harbors a methylation modification that suppresses the expression of its downstream gene, *GH_A07G0948*. In contrast, no DNA methylation was detected in the promoter region, whereas m^5^C methylation occurred in the gene body of *GH_A07G0948* in the Yuc accession, resulting in unrepressed expression (Figure 5d). Genotyping of the SV in (XLZ61 × Yuc) F_2_ revealed that individuals with the XLZ61 type had significantly longer fibers than those with the Yuc type (Figure 5e). Further HiFi sequencing of five cultivated cottons and six semi‐wild cottons revealed a consistent 1,497 bp insertion in the promoter region of *GH_A07G0948* across all cultivated cottons. Notably, this insertion was also detected in partially domesticated semi‐wild accessions (Figure S34a, Supporting Information) but was absent in the ancestral diploid cotton (Figure S35, Supporting Information), which confirmed that *GH_A07G0947* is indeed a novel gene acquired through a post‐domestication insertion event. Expression analysis demonstrated that *GH_A07G0948* was significantly suppressed in accessions harboring this SV (Figure S34b, Supporting Information), which supports the role of the SV‐mediated “acquired methylation” in the regulation of gene expression. We also observed expression differences primarily caused by DNA methylation differences in the gene *GH_A07G1036* (which encodes a pentatricopeptide repeat protein). In XLZ61, CG methylation was decreased upstream of *GH_A07G1036*, leading to a significant increase in its expression (Figure S36a, Supporting Information). Similarly, an example of expression difference primarily caused by RNA methylation differences such as with *GH_A07G1046* (which encodes the 3‐ketoacyl‐CoA synthase protein). In XLZ61 material, *GH_A07G1046* transcripts are subject to m^5^C and m^6^A modification, which leads to a significant increase in their expression (Figure S36b, Supporting Information). Together, these findings highlight the important roles of SV and 3D genome variations in orchestrating DNA/RNA methylation in the course of fiber length domestication.

## 3 Discussion

### 3.1 The Molecular Mechanisms Underlying the Phenotypic Relevance of Inversions

**Figure 6:**
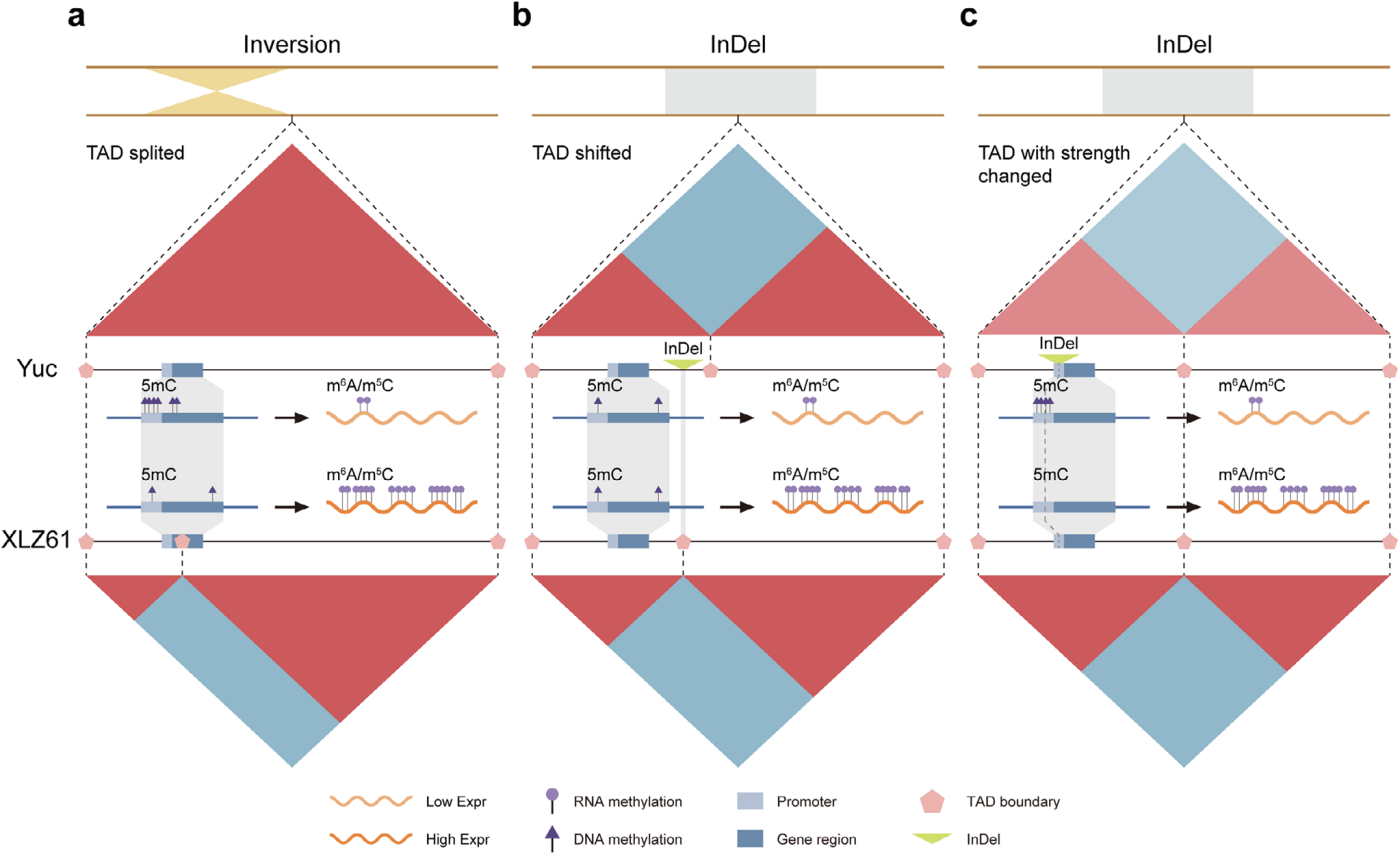
A cooperative model wherein structural/3D genomic variations and methylation changes jointly potentiated cotton fiber domestication. a) The inversion led to a shift in the TAD boundary, transforming the gene from a state of high DNA methylation and low RNA methylation in Yuc to low DNA methylation and high RNA methylation in XLZ61, thereby increasing its expression. b) The InDel caused alterations to the TAD, shifting the gene from a state of low RNA methylation in Yuc to high RNA methylation in XLZ61, resulting in increased expression. c) The InDels deleted a methylated segment in the promoter of genes in XLZ61, shifting the gene from a state of high DNA methylation and low RNA methylation in Yuc to low DNA methylation and high RNA methylation in XLZ61, thereby increasing its expression.

With advancements in genomics, cotton genome assemblies have progressed from draft genomes^[^ ^47^, ^49^, ^50^, ^51^ ^]^ to T2T complete genomes.^[^ ^52^, ^53^, ^54^ ^]^ For *G. hirsutum* in particular, genomes have been successfully assembled for all semi‐wild species and dozens of cultivated varieties.^[^ ^34^, ^35^, ^54^, ^55^, ^56^, ^57^, ^58^, ^59^, ^60^, ^61^, ^62^ ^]^ These genomes have revealed the critical roles of SVs in cotton photoperiod response,^[^ ^35^ ^]^ fiber domestication,^[^ ^34^, ^55^ ^]^ and interspecific differentiation.^[^ ^56^, ^58^ ^]^ However, the genomic information of truly wild species is not yet available. Here, we assembled genomes for the wild species Yuc and the elite accession XLZ61 using HiFi reads and Hi‐C data. A total of 14 inversions larger than 1 Mb were identified and unevenly distributed across 26 chromosomes. The largest inversion (≈32 Mb), in A06, was found in five semi‐wild cottons and cultivated varieties, being absent in the HPF17 and Richmondi (Figure S29, Supporting Information), indicating that this SV locus was likely actively selected during early domestication. Cheng et al. (2023)^[^ ^34^ ^]^ previously reported this largest inversion to be significantly associated with lint percentage and fiber uniformity. However, the mechanism by which inversions affect agronomic traits remains unknown. In this study, we found that inversions can induce sequence variations and remodel the 3D genome, thereby changing the degree of DNA or RNA methylation, which subsequently regulates gene expression and ultimately influences phenotypic variation. For example, the *GH_A06G1342* gene is located at a differential TAD boundary. In the XLZ61, decreased DNA methylation and increased RNA methylation levels collectively lead to its upregulated expression. This gene encodes a ribonuclease H‐like domain protein, which has been demonstrated in rice to regulate grain size through the brassinosteroid signaling pathway.^[^ ^63^ ^]^ Thus, we hypothesize that *GH_A06G1342* may similarly modulate cotton seed size and consequently lint percentage via hormone‐mediated pathways in cotton. These multi‐omics insights provide novel perspectives for understanding the regulatory role of inversions in cotton fiber domestication (**Figure** **6**a).

### 3.2 SVs‐Mediated DNA and RNA Methylation Changes Drove Cotton Fiber Domestication

Beyond SVs, epigenetic mechanisms such as TAD structure, DNA methylation, histone modifications, and RNA methylation can also regulate phenotypic plasticity through facilitating the dynamic control of gene expression without altering DNA sequences.^[^ ^22^, ^23^, ^24^, ^25^, ^26^ ^]^ For example, both DNA methylation and RNA methylation have been shown to regulate the photoperiod response in cotton.^[^ ^36^, ^37^ ^]^ It is known that SVs can significantly promote 3D structural variation;^[^ ^19^, ^20^ ^]^ however, how SVs or 3D genomic variations coordinate with methylation alterations to contribute to cotton fiber domestication remains unclear.

We found that SV‐mediated differential methylation regions or acquired methylation regions can alter gene expression to a greater extent compared to epigenetic modifications alone, implying the SV‐mediated methylation divergence to potentially have greater impacts on phenotypic variations. Subsequently, genomic analyses of the *qFL/A07* locus revealed 76 SVs and four altered TADs. The epigenetic changes associated with these variations included RNA methylation in nine genes, DNA methylation in three genes, and combinatorial genetic‐epigenetic alterations in three genes. These findings support a cooperative model wherein structural/3D genomic variations and methylation divergence jointly potentiated fiber length domestication (Figure 6).

## 4 Experimental Section

### Plant Materials

Fresh leaves were collected for genome sequencing from Yuc and XLZ61 planted in Sanya, Hainan Province, China (18.395 N, 109.174 E). For transcriptome sequencing, 11 types of tissues were sampled from Yuc and XLZ61, including leaf and ovules at −1, 0, 1, 3, 5 DPA, fibers at 10, 15, 20, 25, 30 DPA. For HiFi sequencing, fresh leaves of Yuc and XLZ61 were collected. For Hi‐C sequencing, fibers at 10 and 20 DPA of Yuc and XLZ61 were collected. For ONT direct RNA and Bisulfite sequencing, leaves and fibers at 10 and 20 DPA of Yuc and XLZ61 were collected. For population resequencing, fresh leaves of 300 individuals were collected randomly from the (XLZ61 × Yuc) F_2_ population.

### Illumina Paired‐End Sequencing

The high‐molecular‐weight DNA was isolated from seedlings using a modified cetyltrimethylammonium bromide  extraction method^[^ ^64^ ^]^ and measured using a Qubit fluorescence photometer for DNA concentration and 1% agarose gel electrophoresis for DNA integrity. A total of 200 ng DNA per sample was used as input material for sample preparation. Then, the Watchmaker DNA Library Prep Kit with Fragmentation (CAS: 7K0019‐096) was used to prepare the library. Libraries with 350‐bp inserts were sequenced on the DNBSEQ‐T7 platform, using DNBSEQ‐T7RS High Throughput Sequencing Kit (FCLPE150) V3.0.

### Hi‐C Library Construction and Sequencing

Hi‐C libraries were constructed using XLZ61 and Yuc fibers at 10 DPA and 20 DPA as input, following the standard protocol described previously with some modifications.^[^ ^65^ ^]^ The DNA ends were labeled with biotin‐14‐dCTP and performed blunt‐end ligation of the cross‐linked fragments. Hi‐C sequencing libraries were amplified by PCR (12–14 cycles) and sequenced on the Illumina HiSeq 2500 platform (PE 150 bp).

### PacBio HiFi Sequencing

Libraries for PacBio HiFi sequencing were constructed following the standard protocols of Pacific Biosciences. In brief, high‐molecular‐weight genomic DNA was sheared to a 20 Kb target size, followed by damage repair and end repair, blunt‐end adaptor ligation, and size selection.

### Bisulfite‐Seq

A total amount of 100 ng genomic DNA spiked with 0.5 ng lambda DNA was fragmented by sonication to 200–300 bp with Covaris S220. These DNA fragments were treated with bisulfite using the EZ DNA Methylation‐GoldTM Kit (Zymo Research), and the library was constructed by Novogene Corporation (Beijing, China). Subsequently, pair‐end sequencing of the sample was performed on the Illumina platform (Illumina, CA, USA). Library quality was assessed on the 5400 Fragment Analyzer System. The library was sequenced on the Illumina Novaseq platform. Image analysis and base calling were performed with the Illumina CASAVA pipeline, and finally generated 150 bp paired‐end reads.

### Direct RNA Sequencing

Library preparation and direct RNA sequencing were performed using the SQK‐RNA002 kit (Oxford Nanopore Technologies). Polyadenylated RNA was ligated to the RNA adapter (RTA) with T4 RNA ligase, followed by motor protein (RMX) binding to enable nanopore translocation. The library was purified using AMPure XP beads, eluted in EB buffer, and loaded onto a primed PromethION flow cell with Running Buffer (RB). Sequencing was carried out on the PromethION platform via Guppy software (v5.1.13) (https://nanoporetech.com/zh/document/Guppy‐protocol) for up to 72 h, yielding raw signals for direct RNA analysis without amplification. For each sample, two biological replicates were performed.

### RNA‐Seq Library Construction and Sequencing

RNA‐seq libraries were constructed using the Illumina TruSeq Stranded RNA Kit (Illumina, San Diego, CA, USA) and were sequenced on the Illumina NovaSeq platform. For each sample, three biological replicates were performed.

### Genome Assembly

HiFi reads were sequenced on the PacBio Sequel II platform, and quality control was performed using CCS software (https://github.com/PacificBiosciences/ccs) (v.6.0.0) with the parameters “min‐passes = 3, min‐rq = 0.99.” The contigs of Yuc and XLZ61 were then assembled using Hifiasm (0.16.1)^[^ ^66^ ^]^ using quality‐controlled reads. The Hi‐C data were used to cluster, orient, and sort the contigs using HapHiC (v 1.0.5)^[^ ^67^ ^]^ to obtain a near chromosome‐level assembly, and Juicebox (v.1.11.08)^[^ ^68^ ^]^ was then used to manually correct the genome according to the intensity of chromosome interactions to obtain the final chromosome‐level genome.

### Validation of the Yuc and XLZ61 Assemblies

The accuracy of the Yuc and XLZ61 assemblies was estimated from mapped K‐mers via Merqury (v.1.1).^[^ ^39^ ^]^ To measure genome coverage based on read‐mapping rates, Illumina short reads and HiFi reads were mapped against the assembled genome sequences.

### Identification of Chromosomal Genomic Variations

Whole genome alignment of TM‐1 and XLZ61, TM‐1 and Yuc genomes was performed using the minimap2 (v2.16)^[^ ^69^ ^]^ program with parameters of “‐ax asm5 –eqx”. Then, SyRI software (v1.5)^[^ ^41^ ^]^ was used to identify structural variations and syntenic regions. All variants were annotated using ANNOVAR software.^[^ ^70^ ^]^

### SV Genotyping

The SVs (inversions, insertions, and deletions) were merged using SURVIVOR (v 1.0.7)^[^ ^42^ ^]^ software with default parameters. The merged SVs were genotyped for the 300 F_2_ accessions using paragraph software (v2.4a).^[^ ^71^ ^]^

### Transcriptome Analysis

The Illumina clean RNA‐seq reads were mapped to the reference genome of TM‐1^[^ ^30^ ^]^ using HISAT2 (v 2.1.0) with default settings.^[^ ^72^ ^]^ High‐quality mapping reads were used to calculate gene expression levels using StringTie (v2.1.4)^[^ ^73^ ^]^ with parameter settings (–fr ‐e ‐G).

### Analysis of Differentially Expressed Genes

The DEGs were identified using DESeq2 (v1.26.0)^[^ ^74^ ^]^ with the threshold of 2‐fold expression changes | log_2_(fold‐change) | ≥ 1 and false discovery rate (FDR) < 0.05.

### Hi‐C Data Processing

The HiC‐Pro (v3.1.0) pipeline^[^ ^75^ ^]^ was used to obtain Hi‐C contact read pairs. First, the low‐quality reads and adaptors were removed using fastp (v 0.21.0).^[^ ^76^ ^]^ Then, the clean data was mapped to the TM‐1 genome using Bowtie2 (v 2.5.4),^[^ ^77^ ^]^ and multiple hits, singleton, dangling end, self‐circle, low MAPQ, and PCR duplicates were removed. An iterative correction and eigenvector decomposition (ICE) method was applied to normalize the Hi‐C contact matrices at resolutions of 5, 20, 40, and 500 Kb.^[^ ^75^ ^]^

### A and B Compartments Identification

The hicPCA program embedded in HiCExplorer^[^ ^78^ ^]^ was used to delineate A/B compartments at the 40 Kb resolution. Bins with the first principal component values greater than zero and having a high gene density and low DNA methylation on the chromosome level were regarded as A compartment; bins with values less than zero and having a low gene density and high DNA methylation were regarded as B compartment. If the values of at least two consecutive bins changed from positive to negative, these bins represent switching from the A compartment to the B compartment, and vice versa represent switching from the B compartment to the A compartment.

### Topologically Associated Domain Structure Identification

TAD structures and differential TAD structures were identified using the hicFindTADs and hicDifferentialTAD programs embedded in HiCExplorer (v3.7.5),^[^ ^78^ ^]^ respectively, at the 20 Kb resolution.

### DRS Data Handling and Gene Structure Analysis

The DRS fastq reads for each sample were filtered using Nanofilt (v2.5.0)^[^ ^79^ ^]^ with options ‐l 100 ‐q 7. The full‐length reads were aligned to the genome with minimap2 (v2.16)^[^ ^40^ ^]^ and then were corrected and collapsed with flair (v1.7).^[^ ^80^ ^]^ Structure annotation was performed using Gffcompare (v0.12.6) (https://github.com/gpertea/gffcompare) to identify known and novel transcripts. Unmapped transcripts and structured annotated transcripts functional annotation.

### Quantification of Transcriptional Expression

Full‐length DRS reads were aligned to the transcriptome using minimap2 (v2.16).^[^ ^40^ ^]^ Transcripts were quantified using salmon (v0.14.1)^[^ ^81^ ^]^ based on the alignment files, parameters quant ‐noErrorModel ‐p 4 ‐t ref_transcript.fa ‐l U ‐a sample.bam. Transcript counts were normalized and used to calculate TPM to characterize the transcript expression.

### Differential Expression Analysis

Differential expression analysis of two conditions/groups (two biological replicates per condition) was performed using the DESeq2 R package (v1.26.0).^[^ ^74^ ^]^ DESeq2 provides statistical routines for determining differential expression in digital gene expression data using a model based on the negative binomial distribution. The resulting *P*‐values were adjusted using Benjamini and Hochberg's approach for controlling the false discovery rate. Genes with an adjusted *P*‐value < 0.05 found by DESeq2 were assigned as differentially expressed.

### RNA Methylation

RNA methylation and motif analysis Tombo (v.1.5.1) (https://github.com/nanoporetech/tombo), a suite of tools officially released by the Nanopore community, was designed for identifying modified nucleotides directly from raw nanopore sequencing data (FAST5 files containing electrical signals). In alternative mode, Tombo can predict m^5^C sites in Direct RNA sequences, with the top 1000 loci exhibiting the highest alternate proportion (defined as the ratio of reads supporting methylation to total reads covering the site) selected for downstream motif analysis and statistics. The MINES pipeline extends Tombo's de novo mode with additional computational steps to predict m^6^A modifications in Direct RNA sequences. Here, alternate proportion refers to the fraction of reads supporting methylation at a given genomic position. For motif analysis, a 5‐bp sequence flanking each methylation site was extracted, and conserved 5‐mer sequences shared across multiple sites were identified as motif signatures.

### Differential Methylation Loci Analysis

Differential methylation loci (DML) analysis using methylKit statistical testing.^[^ ^82^ ^]^ The Methylation loci with a difference threshold: ≥20% (Δ methylation level) and q‐value < 0.05 were considered as DML.

### Multiple Testing Correction

Apply the SLIM method (Sliding Linear Model) for *P*‐value adjustment.

### WGBS Analysis

Fastp (v 0.21.0)^[^ ^76^ ^]^ was used to trim the low‐quality reads and adaptors using the default parameters, and the clean data were mapped to the TM‐1 reference genome^[^ ^30^ ^]^ for each accession using Bismark (v2.2.5).^[^ ^83^ ^]^ After filtering out the duplicate reads using “deduplicate_bismark” in the bismark software package, the methylation information for each cytosine site was extracted using “bismark_methylation_extractor” in the bismark software package. Sites covered by >5 mapped reads were only retained to obtain reliable methylation sites. To investigate the chromosome distribution of DNA methylation, the chromosome was divided into windows of 500 Kb length. Then, the methylation level of each window was calculated. To investigate the distribution of DNA methylation franking genes/TEs, the upstream 3 Kb, downstream 3 Kb, and body regions of genes/TEs were extracted, and was divided them evenly into 100 bp windows, respectively. Then, the methylation level of each bin was calculated.

### DMR and AMR Identification

DMRs were identified between comparable groups using the R package MethylKit.^[^ ^82^ ^]^ The genome was tiled into 1 Kb non‐overlapping windows. Windows exhibiting a methylation difference of ≥20% and a statistically significant q‐value of < 0.05 were defined as CG DMRs; windows exhibiting a methylation difference of ≥15% and a statistically significant q‐value of < 0.01 were defined as CHG DMRs; windows exhibiting a methylation difference of ≥10% and a statistically significant q‐value of < 0.01 were defined as CHH DMRs

InDel‐AMRs identification was performed as follows: First, the genome was partitioned into 100 bp windows. The methylation level for each window was computed and retained those with a freqC value greater than 0.2. Subsequently, overlaps between SVs and these methylated windows were identified using BEDTools,^[^ ^84^ ^]^ and regions with an overlap exceeding 90% were defined as InDel‐AMRs.

### GO Enrichment

GO enrichment analyses of the DNA methylation‐related genes were performed using the clusterProfiler^[^ ^85^ ^]^ software.

### SNP Calling

All raw reads were processed for quality control and filtered using fastp (v 0.21.1)^[^ ^76^ ^]^ with default parameters. Clean reads were mapped to the TM‐1 reference genome^[^ ^30^ ^]^ using BWA (v 0.7.17‐r1188) with the parameters of “mem ‐t 20 ‐M ‐R”.^[^ ^86^ ^]^ The mapping results were sorted and duplicates marked using functions implemented in SAMtools (v1.9)^[^ ^87^ ^]^ and Picard (http://broadinstitute.github.io/picard/). Variants for genetic map construction were called using SAMtools mpileup.^[^ ^87^ ^]^ High‐quality SNPs were filtered using the parameters mapping quality >20, genotyping rate >70%. Variants for BSA‐seq analysis were called using GATK (v.4.1.2.0) HaplotypeCaller and GenotypeGVCFs.^[^ ^88^ ^]^ High‐quality SNPs were filtered using the parameters mapping quality >20, genotyping rate = 100%.

### BSA‐Seq Analysis

The R package QTLseqr (v0.7.0)^[^ ^89^ ^]^ was utilized to identify candidate regions with a 2‐Mb window size and a 100 Kb increment.

### High‐Density Genetic Map Construction and QTL Mapping

Polymorphic markers having an aa × bb segregation pattern between the two parental lines were retained for the F_2_ individuals. Recombinant breakpoints were identified using a hidden Markov model (HMM) approach.^[^ ^90^ ^]^ The bins with significantly distorted segregation (*p*‐value < 0.001) were filtered using a chi‐square test, and the ones that remained served as genetic markers and were used to construct the genetic linkage map with IciMapping (v 4.1.0.0).^[^ ^91^ ^]^ The collinearity between the genetic map and physical positions was visualized using ALLMAPS.^[^ ^92^ ^]^ IciMapping^[^ ^91^ ^]^ was also applied to map QTLs related to fiber quality traits. The ICIM method was used to scan the genetic map and estimate the likelihood of a QTL and its corresponding effect at every 1 cM. 1000 permutation tests were conducted to determine empirically derived logarithm of odds (LOD) scores.

### Statistical Methods

The significance was determined by a two‐tailed t‐test using R software.

### Sources of Published Datasets

The TM‐1^[^ ^30^ ^]^ and *Purpurascens* ^[^ ^34^ ^]^ genome and annotation were downloaded from http://cotton.zju.edu.cn/.^[^ ^93^ ^]^ The *Punctatum*,^[^ ^57^ ^]^ *Latifolium*,^[^ ^35^ ^]^ *Paleri*,^[^ ^55^ ^]^ *Morrilli*,^[^ ^55^ ^]^ *Richmondi*,^[^ ^55^ ^]^ *Galante*,^[^ ^55^ ^]^ ZM113^[^ ^54^ ^]^ genomes were downloaded from https://www.cottongen.org/.^[^ ^94^ ^]^

## Conflict of Interest

The authors declare no conflict of interest.

## Author Contributions

L.S. and S.J. contributed equally to this work. L.F. conceived the research project and designed the experiments. L.S., S.J., T.Z., and Y.H. assembled the genomes. L.S., S.J., and Z.S. collected the DNA and RNA samples for sequencing. L.S., S.J., H.J., and T.P. analyzed the bioinformatic data. L.S., S.J., X.G., T.Z., and L.F. participated in writing and revising the manuscript. All authors discussed the results and commented on the manuscript.

## Supporting information



Supporting Information

Supplemental Tables 1‐16

## Data Availability

The Yuc and XLZ61 assembly and annotation are available at http://cotton.zju.edu.cn/ and figshare (https://doi.org/10.6084/m9.figshare.30692423.v1). The genome sequencing data used for Yuc and XLZ61 assembly, including PacBio HiFi data and HiC data was deposited in the China National Genomics Data (https://ngdc.cncb.ac.cn) (PRJCA042871). The raw transcriptomics data was deposited in the China National Genomics Data (PRJCA042872). The WGBS data used for DNA methylation identification was deposited in the China National Genomics Data (PRJCA042873). The ONT DRS data used for RNA methylation identification was deposited in the China National Genomics Data (PRJCA042874). The population resequencing data and BSA‐seq data used for QTL mapping was deposited in the China National Genomics Data (PRJCA042877 and PRJCA052110).
