# Supplementary material for: Structural Variation and 3D Genome‐Driven DNA/RNA Methylation Divergence Contributing to Cotton Fiber Domestication
Source: Adv Sci (Weinh). 2025 Dec 2;13(9):e14381. doi: 10.1002/advs.202514381 (PMC12903982; doi:10.1002/advs.202514381)
Supplement: Supplementary file 1 — Supporting Information [file ADVS-13-e14381-s001.docx]

**Structural variation and 3D genome-driven DNA/RNA methylation divergence contributing to cotton fiber domestication**

Lei Shao^1,2#^, Shangkun Jin^1#^, Haojie Jiang^1^, Tianyu Pan^1,2^, Zesheng Rui^1^, Xiaowen Shi^1^, Ting Zhao^1^, Zhanfeng Si^1^, Xueying Guan^1,2^, Yan Hu^1,2^, Tianzhen Zhang^1,2^, Lei Fang^1,2*^

1. Zhejiang Key Laboratory of Crop Germplasm Innovation and Utilization, Key Laboratory of Speed Breeding in Plant Factory (Ministry of Agriculture and Rural Affairs), College of Agriculture and Biotechnology, Zhejiang University, Hangzhou 310058, China.

2. Hainan Institute of Zhejiang University, Sanya 572025, China

^#^These authors contributed equally to this work.

^*^Correspondence: Lei Fang (fangl@zju.edu.cn)


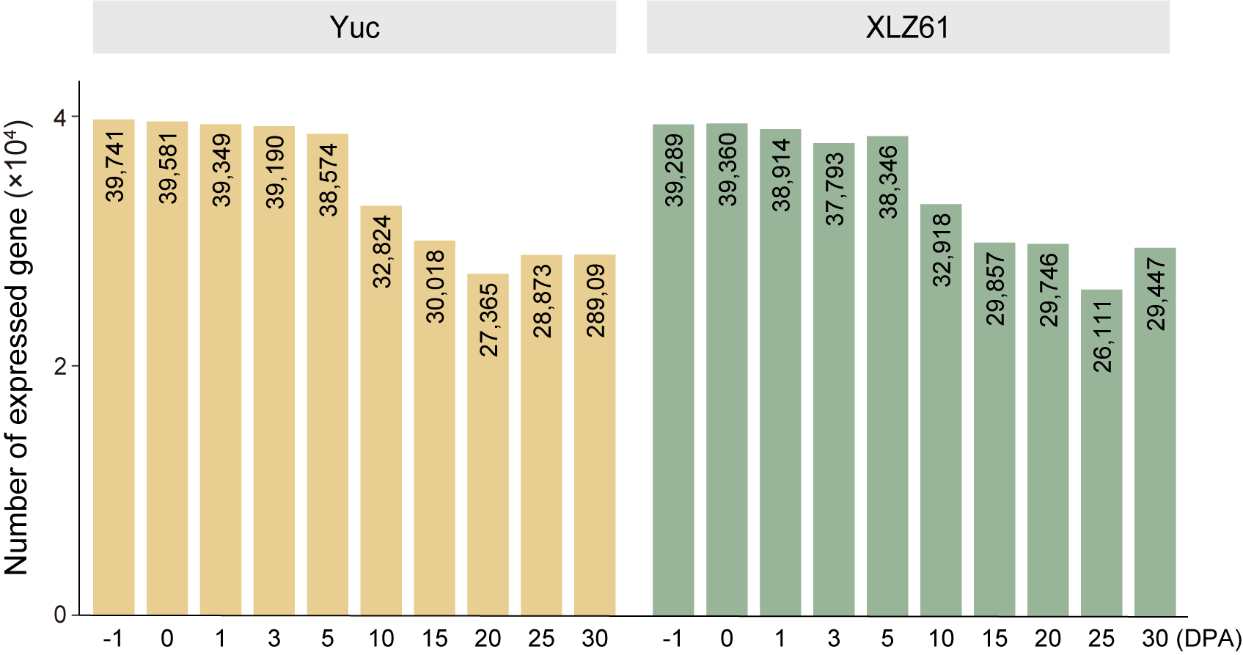


**Figure S1 Summary of expressed genes during fiber development in Yuc and XLZ61.**


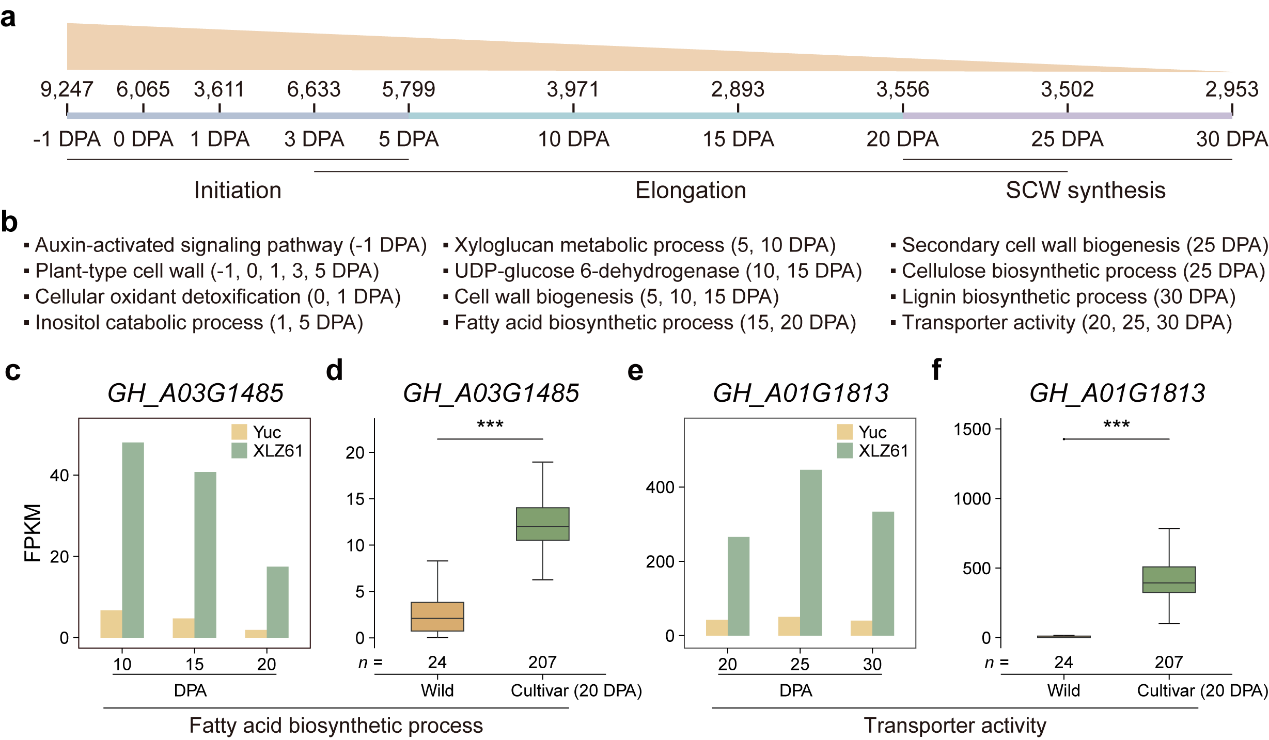


**Figure S2** **DEG numbers and GO enrichment analysis during fiber domestication. a** Number of DEGs at various stages of fiber development. **b** GO enrichment analysis of differentially expressed genes. **c** Expression levels of the *GH_A03G1485* gene in the fatty acid synthesis pathway at 10, 15, and 20 DPA. **d** Expression levels of *GH_A03G1485* across the population. The significance was determined by t-test. *** indicated the *p* <0.001. **e** Expression levels of *GH_A01G1813* in the transporter protein pathway at 20, 25, and 30 DPA. **f** Expression levels of *GH_A01G1813* across the population. The significance was determined by t-test. *** indicated the *p* <0.001.


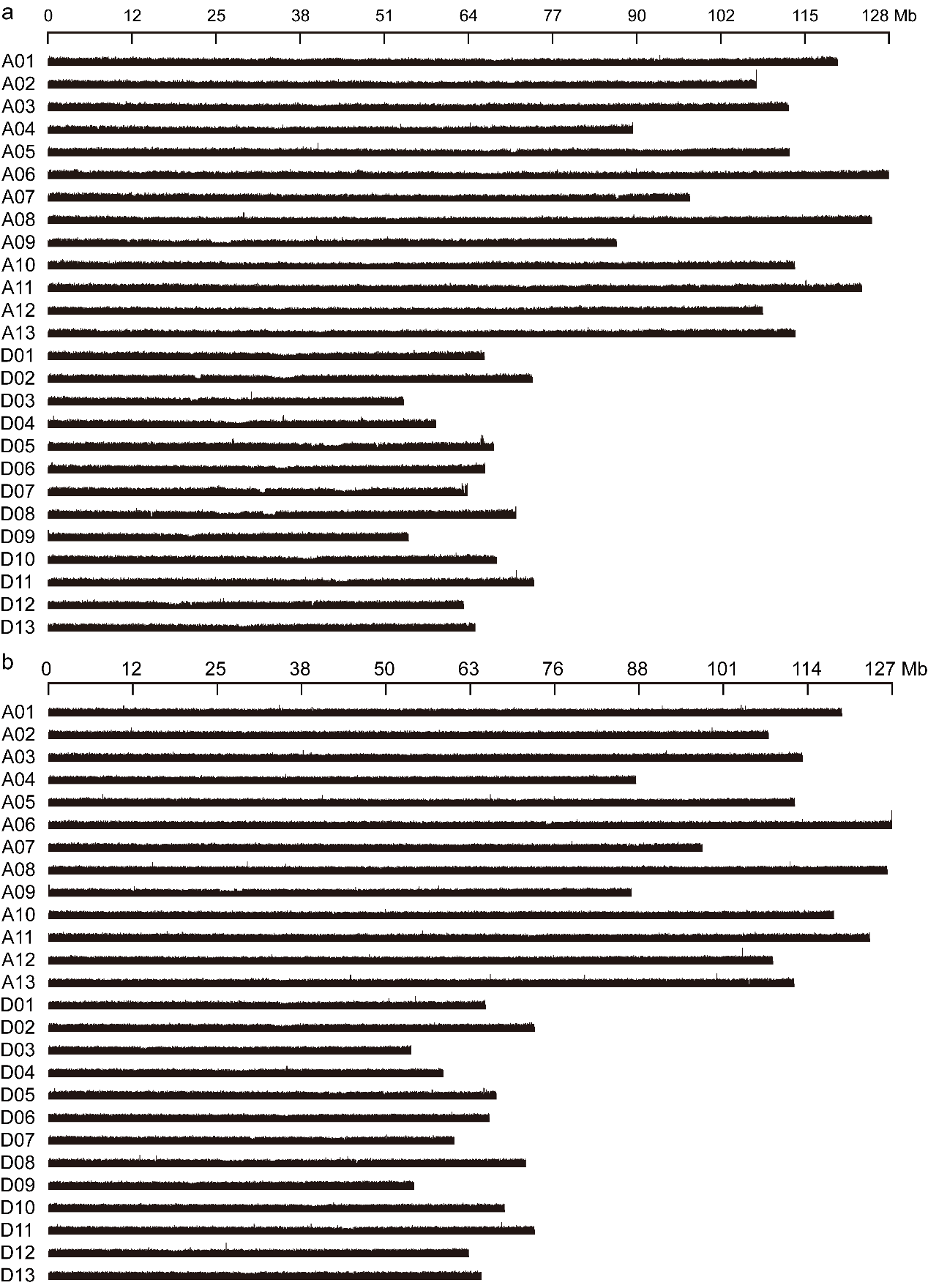


**Figure S3. Whole-genome coverage of HiFi reads in Yuc (A) and XLZ61 (B) genome.** All data are shown in 100 Kb windows.


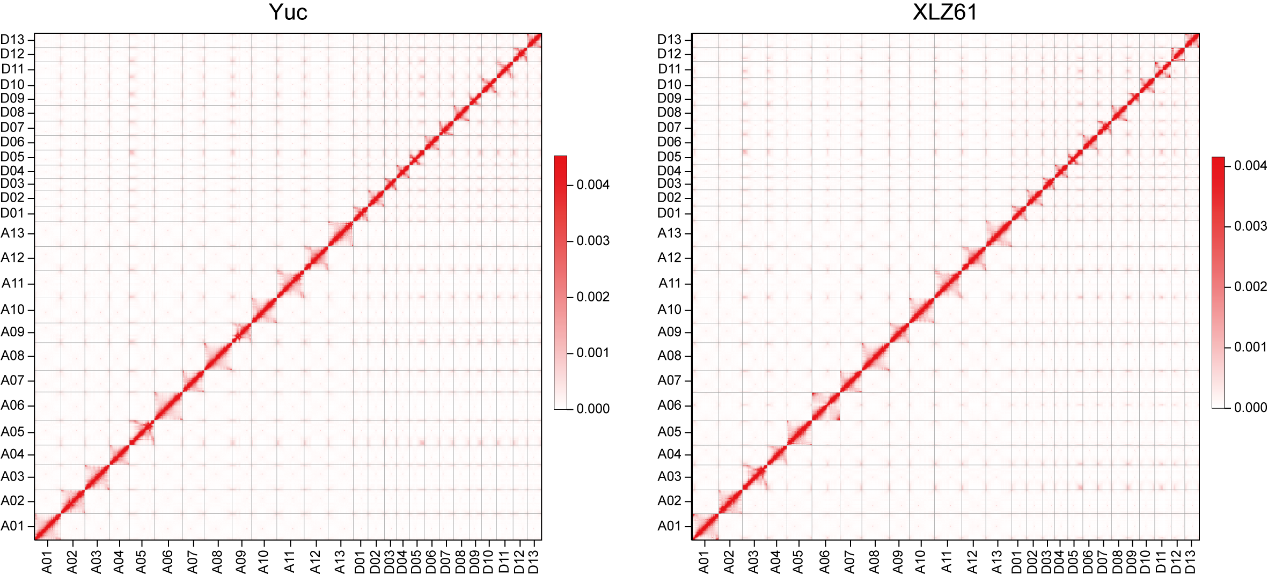


**Figure S4** **Hi-C interaction heatmap of the Yuc (A) and XLZ61 (B) genomes.** Hi-C contact data were mapped on the Yuc and XLZ61 genome, respectively. Strong signals were observed on diagonal region, indicating that the contigs were accurately oriented on the pseudochromosomes.


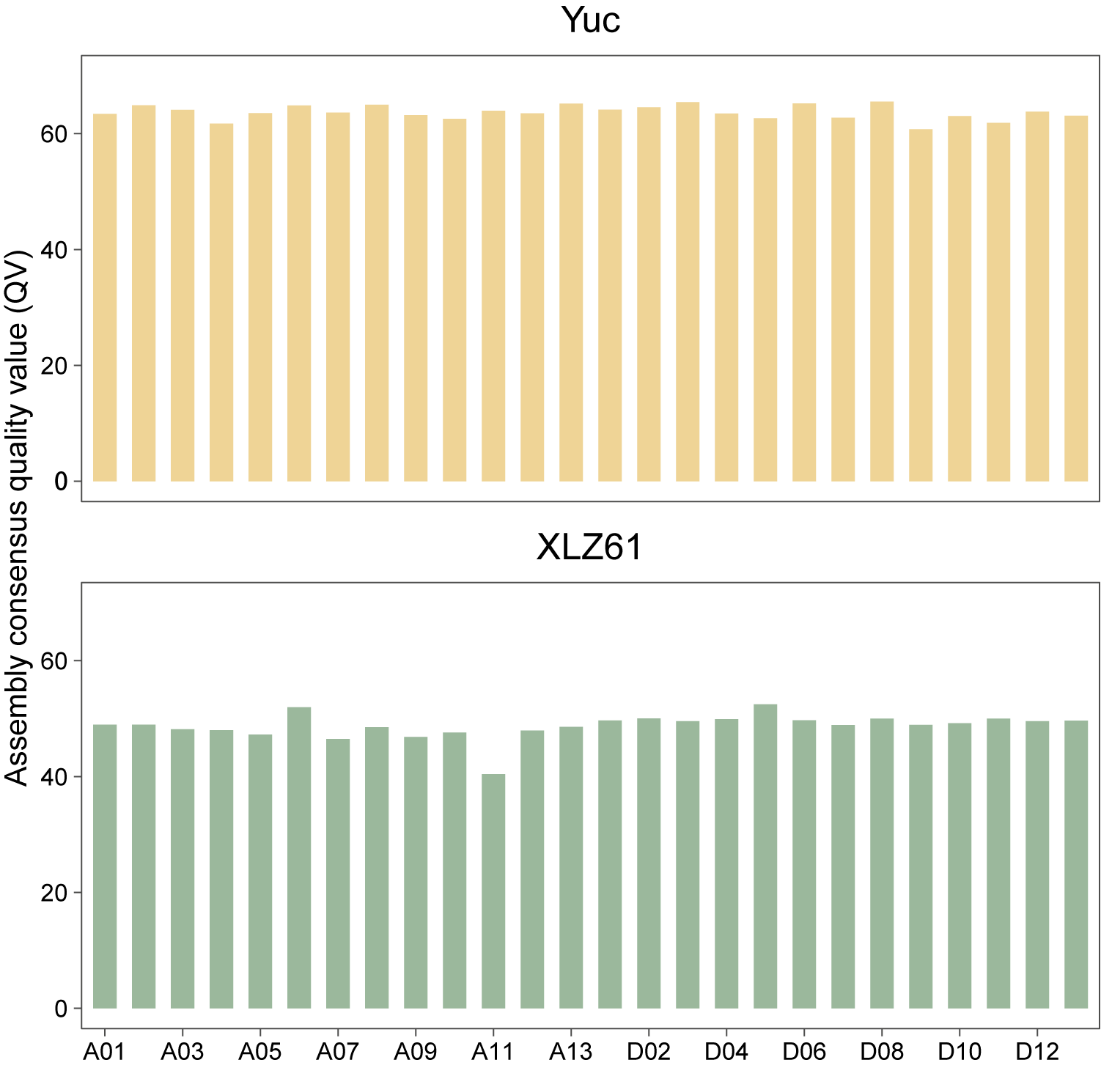


**Figure S5** **Assembly consensus quality value (QV) scores of the individual and collective (All) chromosomes of the Yuc and XLZ61 genomes.**

**
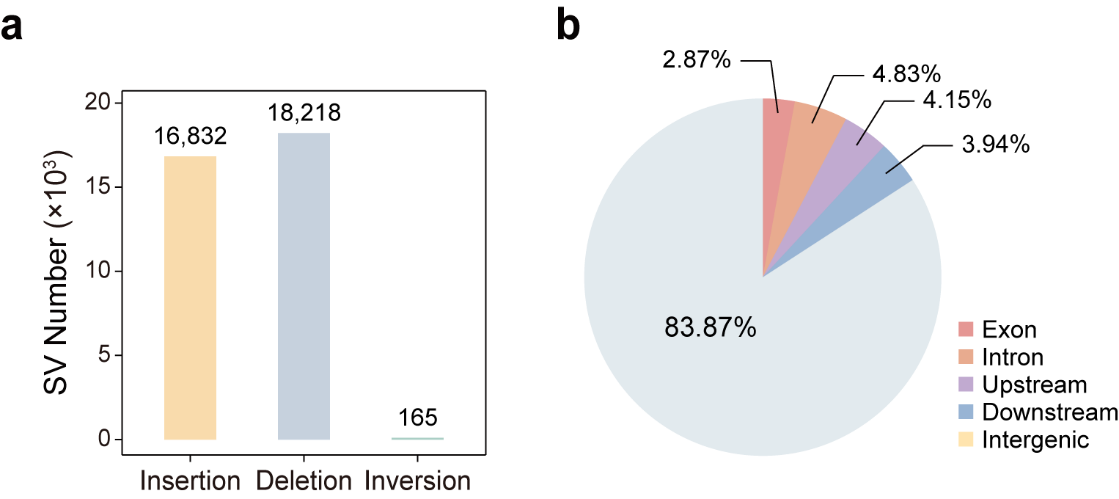
**

**Figure S6** **The number** **structural variations identified between Yuc and XLZ61 genomes (a) and their annotaion (b).**

**
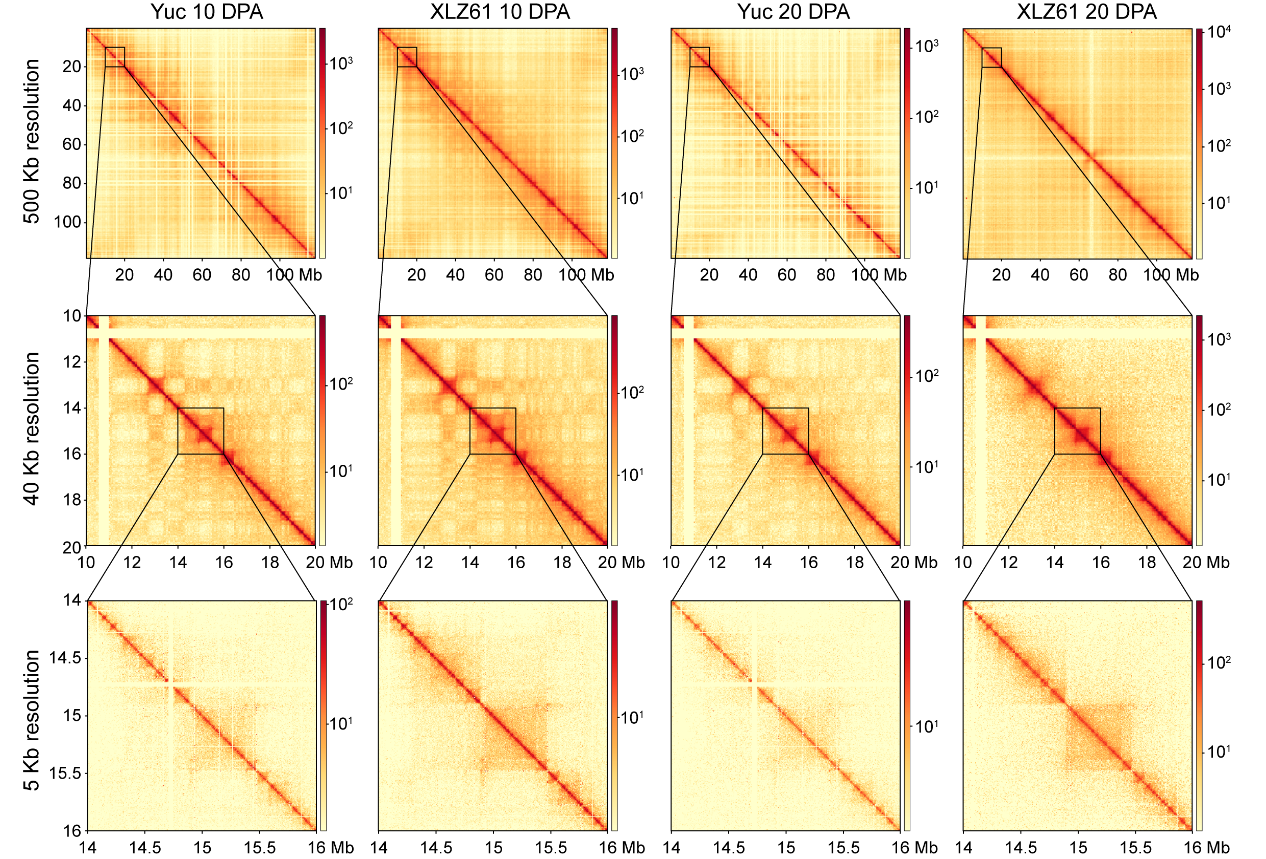
**

**Figure S7** **Multi-resolution Hi-C interaction heatmaps at different stages of fiber development during domestication.** Hi-C matrices were constructed at resolutions of 500 kb (0–118 Mb in chromosome A01), 40 kb (10–20 Mb in chromosome A01), and 5 kb (14–16 Mb in chromosome A01).

**
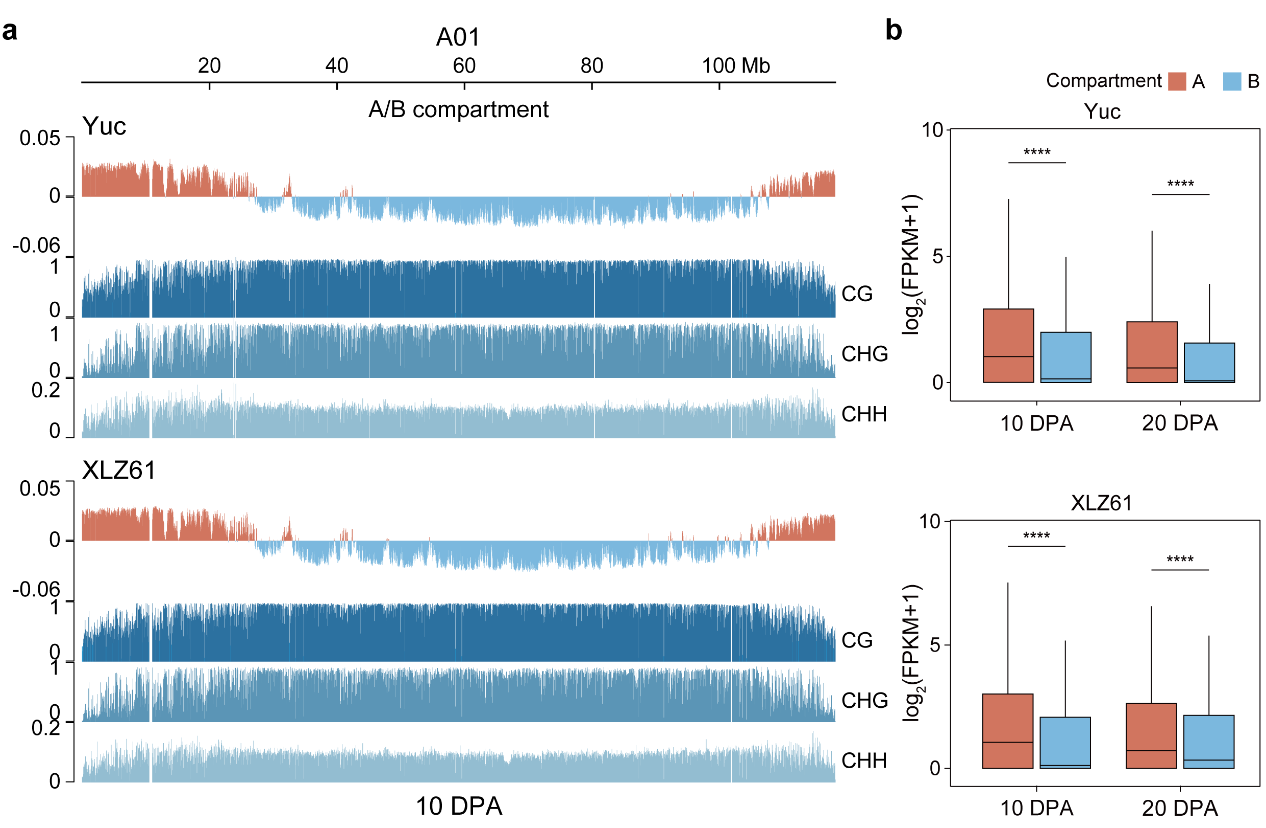
**

Figure S8 The DNA methylation and expression level in A/B compartment. a A/B compartments (40-Kb bins) and DNA methylation (40-Kb bins) throughout chromosome A01. b Comparative analysis of gene expression levels within A/B compartments. The significance was determined by t-test. **** indicated the *p* <0.0001.


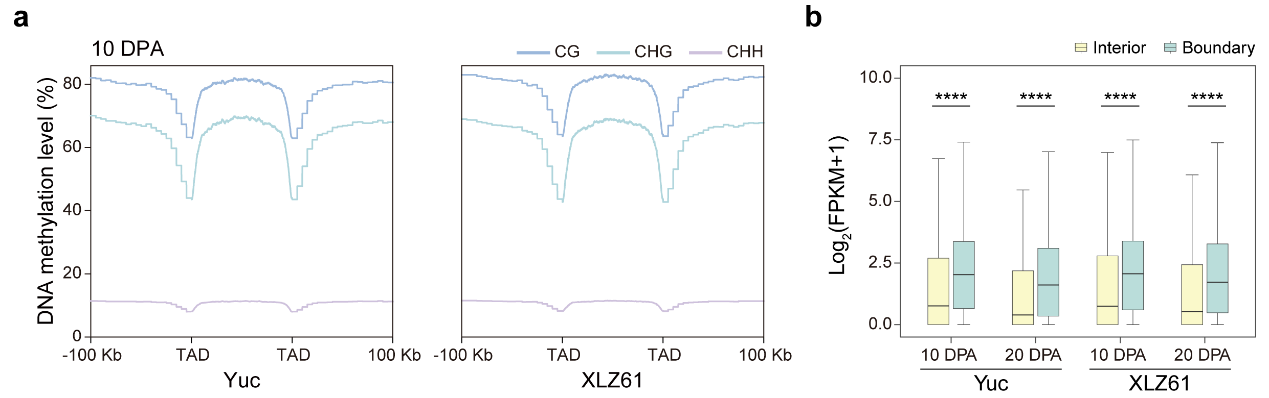


Figure S9 The expression and DNA methylation level in TAD interior/boundary region. a Comparison of the expression level of genes contained in the TAD interior/boundary regions. b Comparison of the DNA methylation level of genes contained in the TAD interior/boundary regions. The significance was determined by t-test. **** indicated the *p* <0.0001.

**
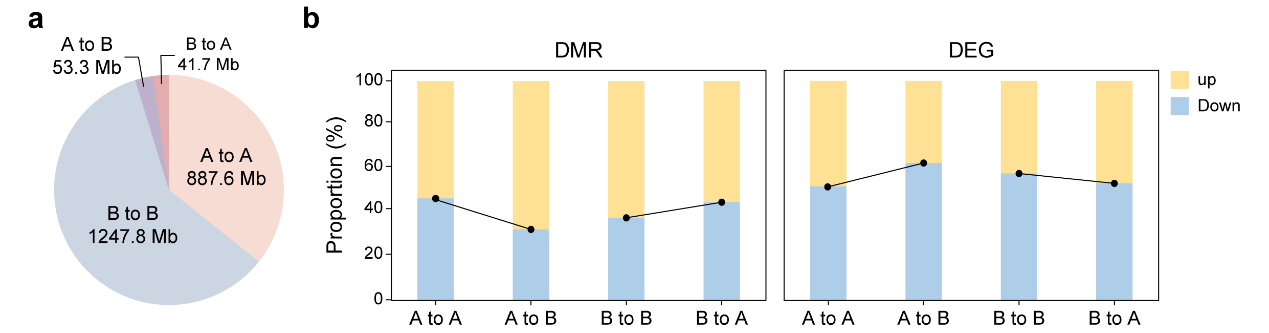
**

**Figure S10 The changes of expression and DNA methylation level in the A/B compartment switching regions. a** The size of the A/B compartment switching regions at 10DPA between Yuc and XLZ61. **b** The number of DEG and DMR in the A/B compartment switching or stable regions.

**
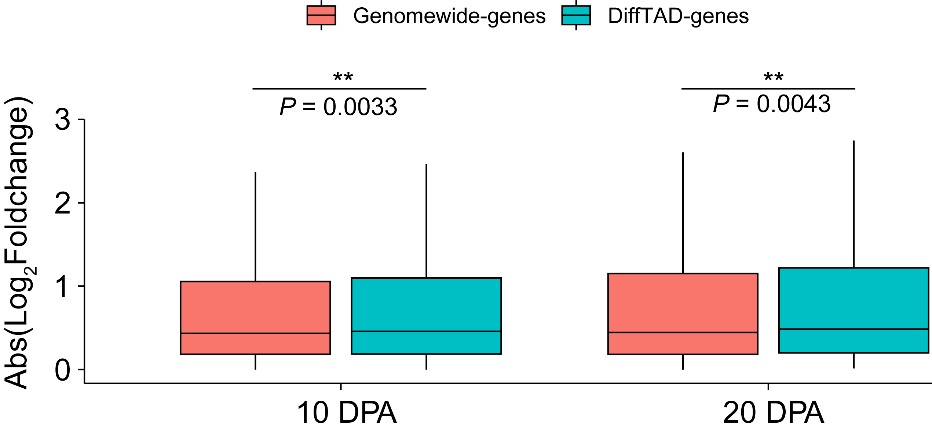
**

**Figure S11. Comparative analysis of gene expression levels with genome-wide and differential TADs.** The significance of fold change of expression was determined by t-test.


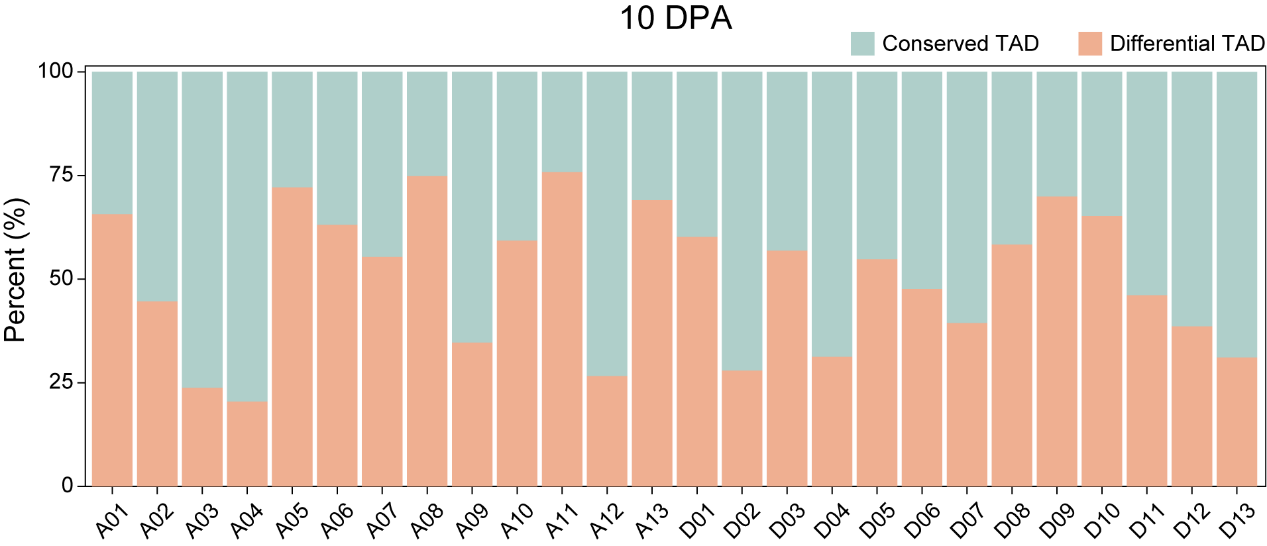


**Figure S12 The percent of differential TAD length across all chromosomes at 10 DPA.**

**
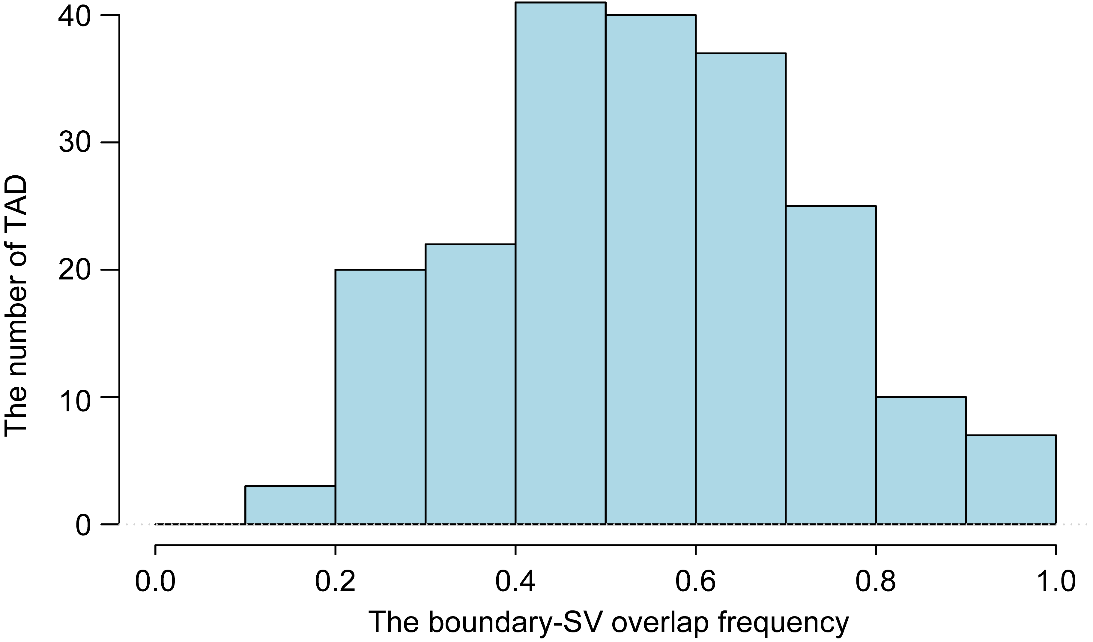
**

**Figure S13 The distribution of SV-TAD boundary coincidence rates across all 10-Mb windows**

**
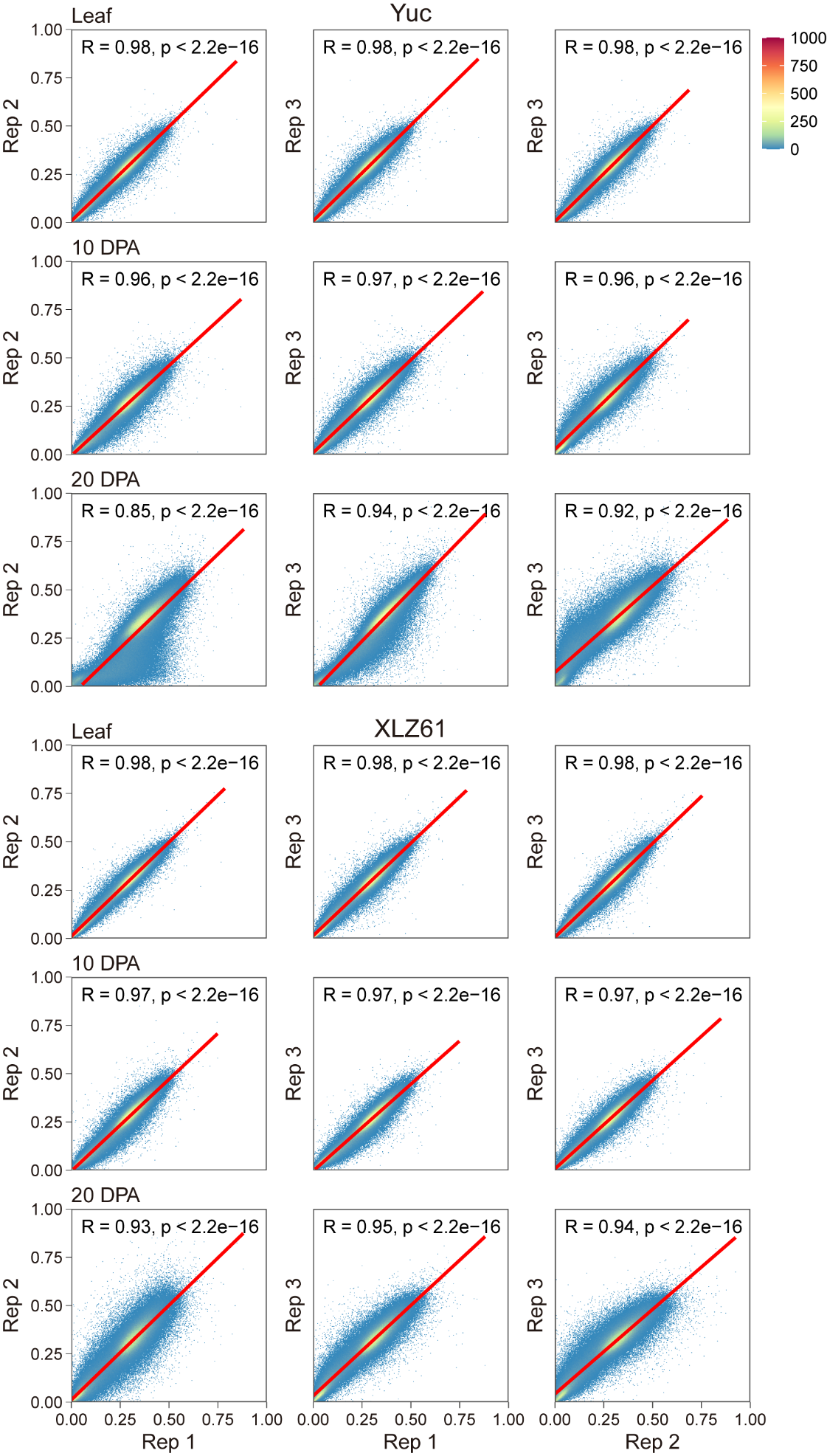
**

**Figure S14 Pearson correlation analysis between three replicates of DNA methylation level in six samples.** R indicates the Pearson correlation coefficient.


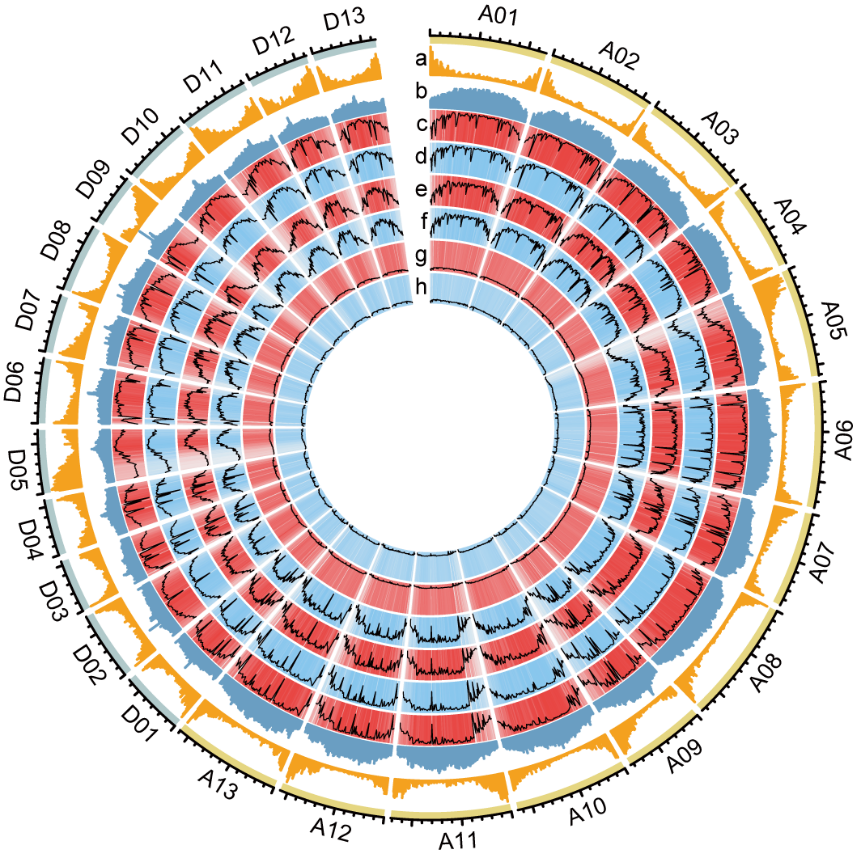


Figure S15 The distribution patterns of DNA cytosine methylation levels in the Yuc and XLZ61 species. a: Density of gene;b: density of TE; c: Density of CG methylation level in XLZ61; d: Density of CG methylation level in Yuc; e: Density of CHG methylation level in XLZ61; f: Density of CHG methylation level in Yuc; g: Density of CHH methylation level in XLZ61; h: Density of CHH methylation level in Yuc.


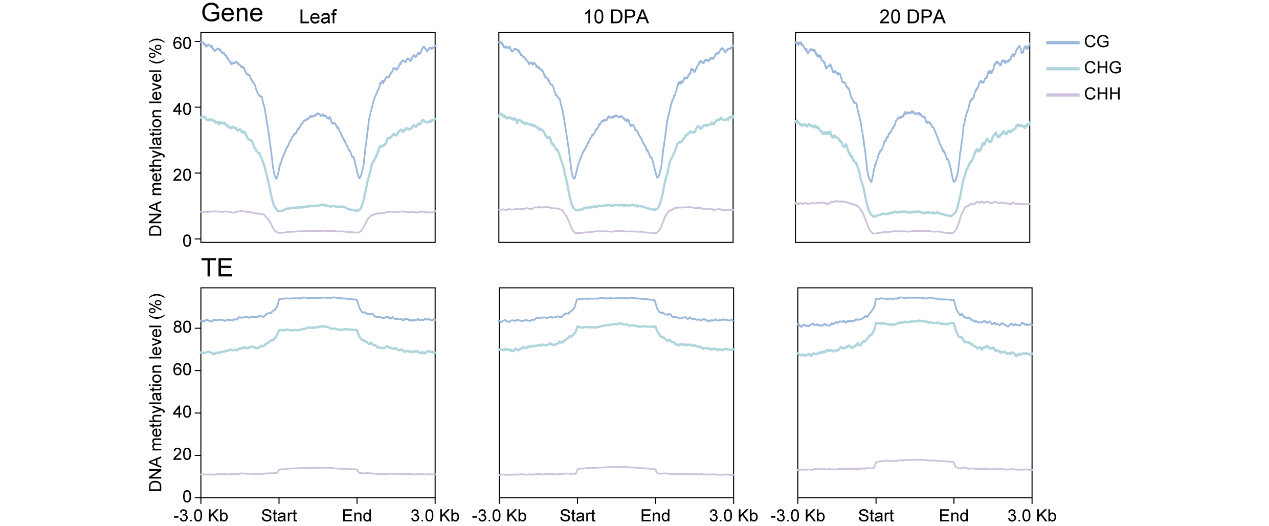


Figure S16 ****DNA Methylation distribution around genes (upper) and transposable elements (TEs) (lower).**** Blue represents CG methylation, orange represents CHG methylation, and green represents CHH methylation.

**
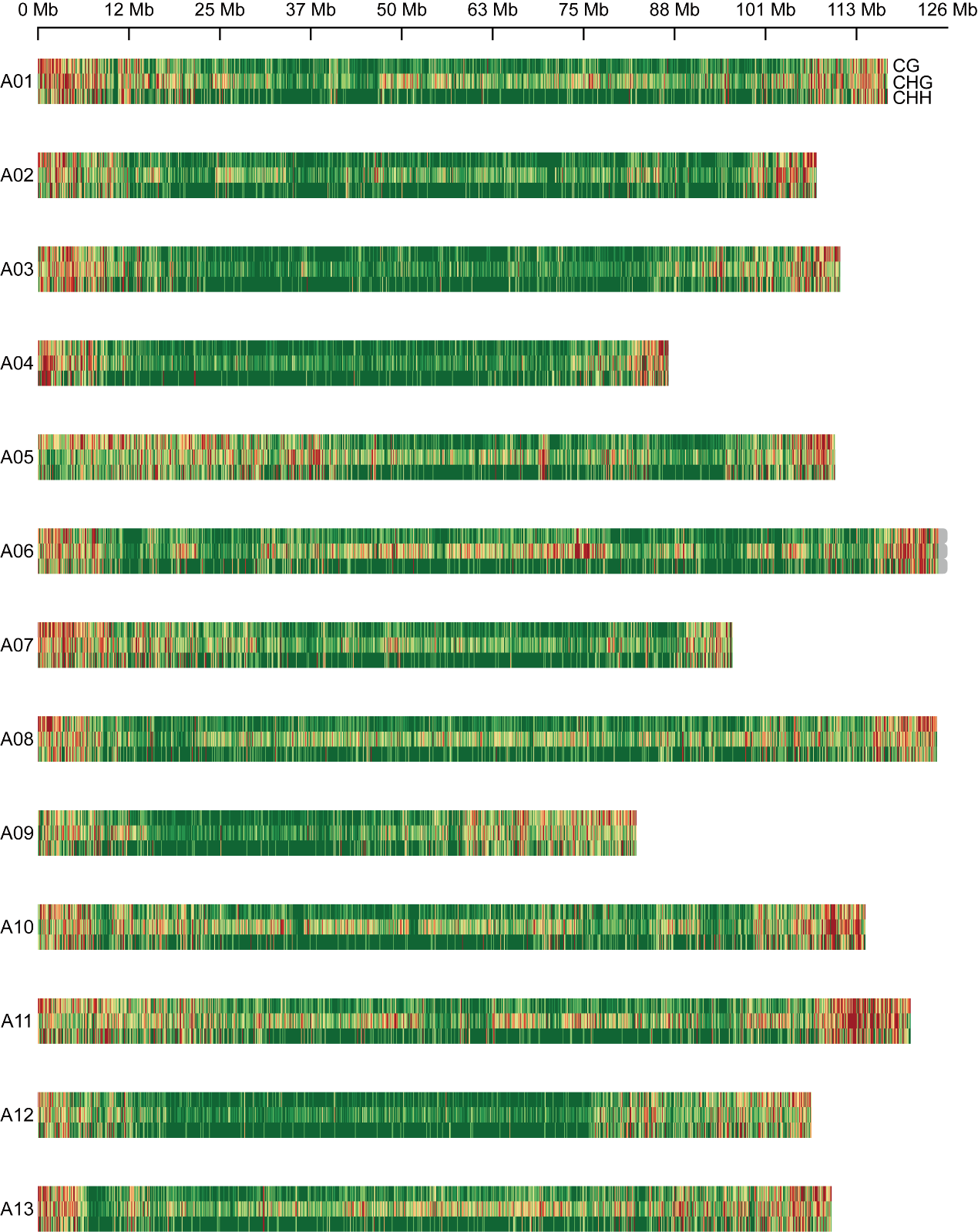
**

**
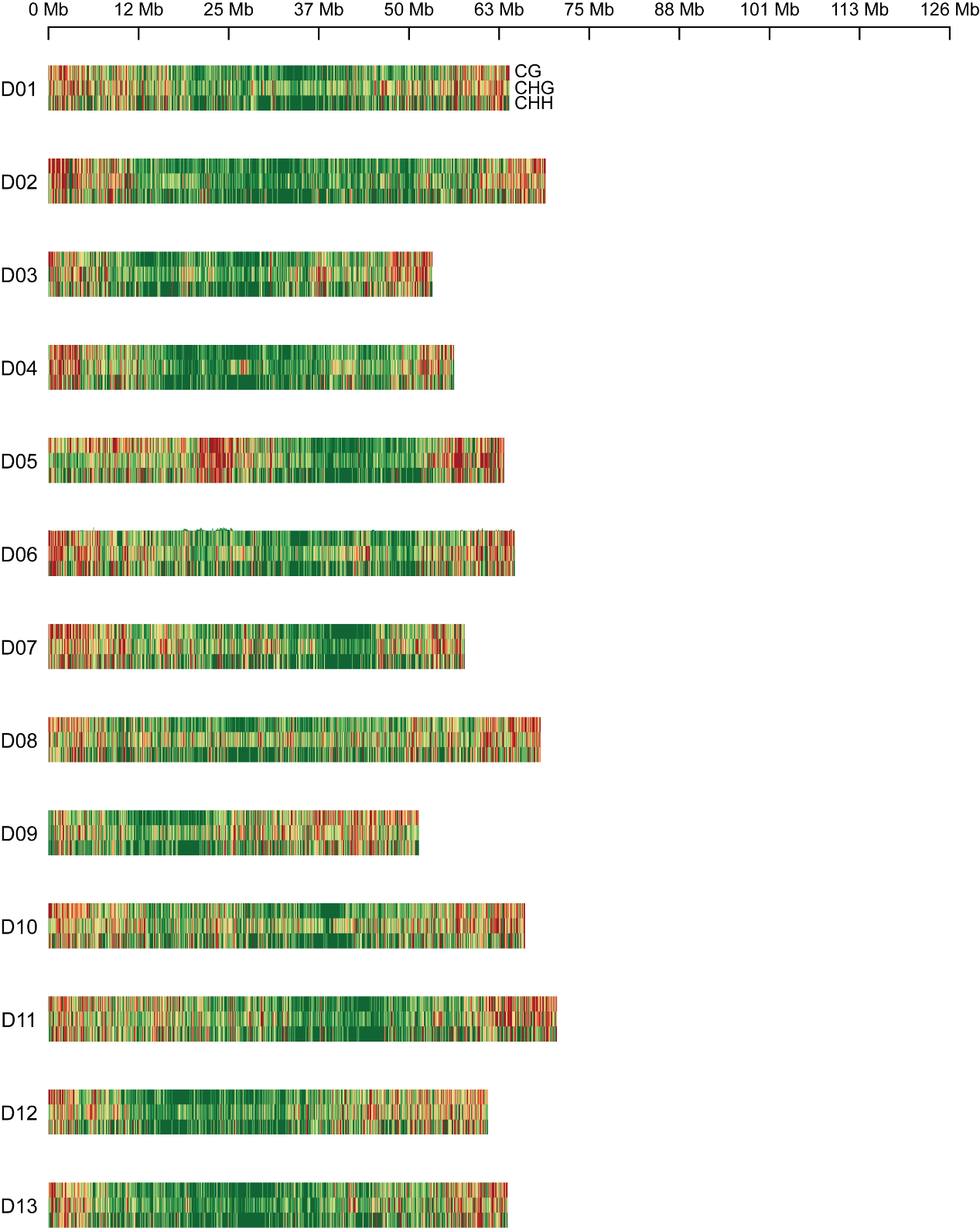
**

**Figure S17 Distribution of differential methylation across the whole genome.** The first layer represents the distribution of differential CG methylation regions, the second layer represents the distribution of differential CHG methylation regions, and the third layer represents the distribution of differential CHH methylation regions.


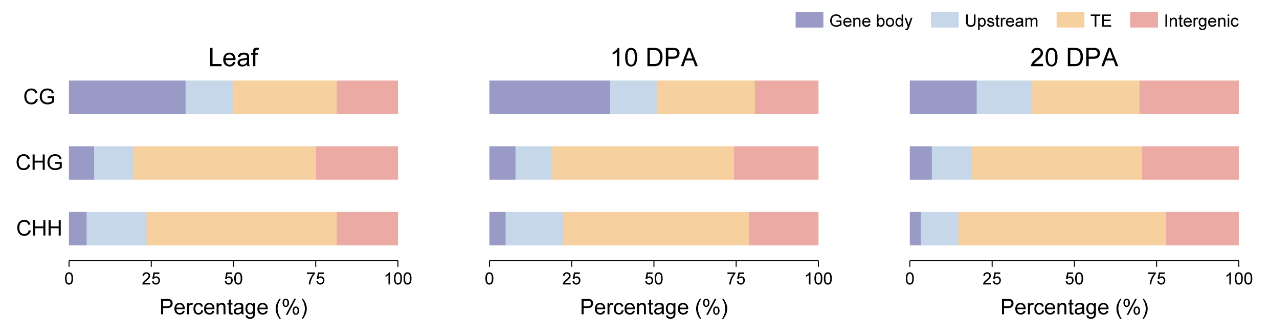


Figure S18 Distribution of DMRs in different genomic regions. The different genomic regions divided into gene body, +2 Kb flanking region (2 Kb upstream of TSS), TEs, and intergenic regions excluding TEs.


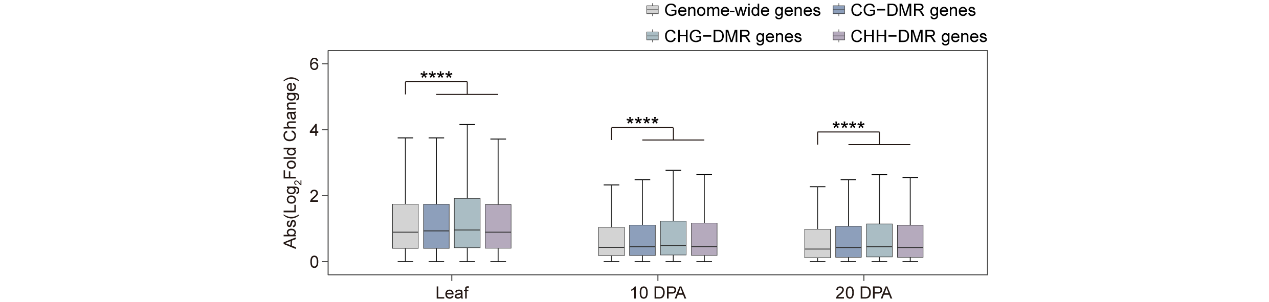


Figure S19 The impact of DMRs in gene expression. Box plot of gene expression changes (absolute values) in DMR-associated proximal genes (DPGs) and genome-wide genes. The significance was determined by t-test. **** indicated the *p* <0.0001.


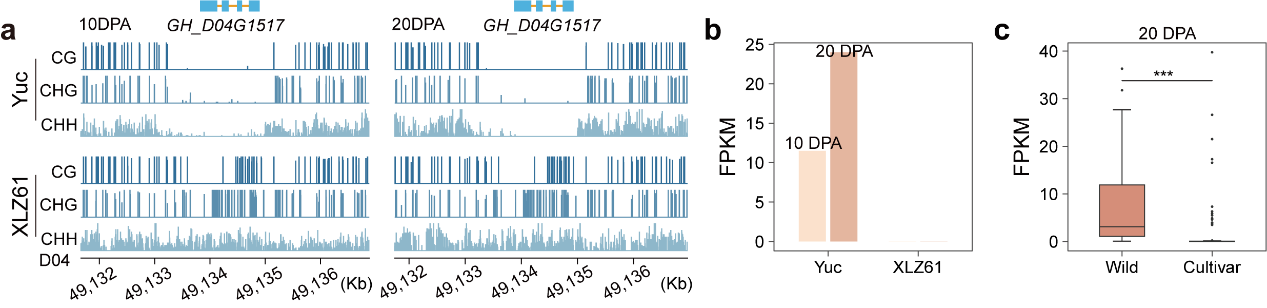


**Figure S20 Methylation-regulated selection of *GH_D04G1517* during cotton fiber development. a** DNA methylation levels in the promoter and gene body regions of *GH_D04G1517* in Yuc and XLZ61 at 10 and 20 DPA. **b** Expression suppression of *GH_D04G1517* in Yuc and XLZ61. **c** Population-level expression analysis of *GH_D04G1517* in cultivated (*n* = 207) versus wild/semi-wild cotton species (*n* = 24). The significance was determined by t-test. *** indicated the *p* <0.001.


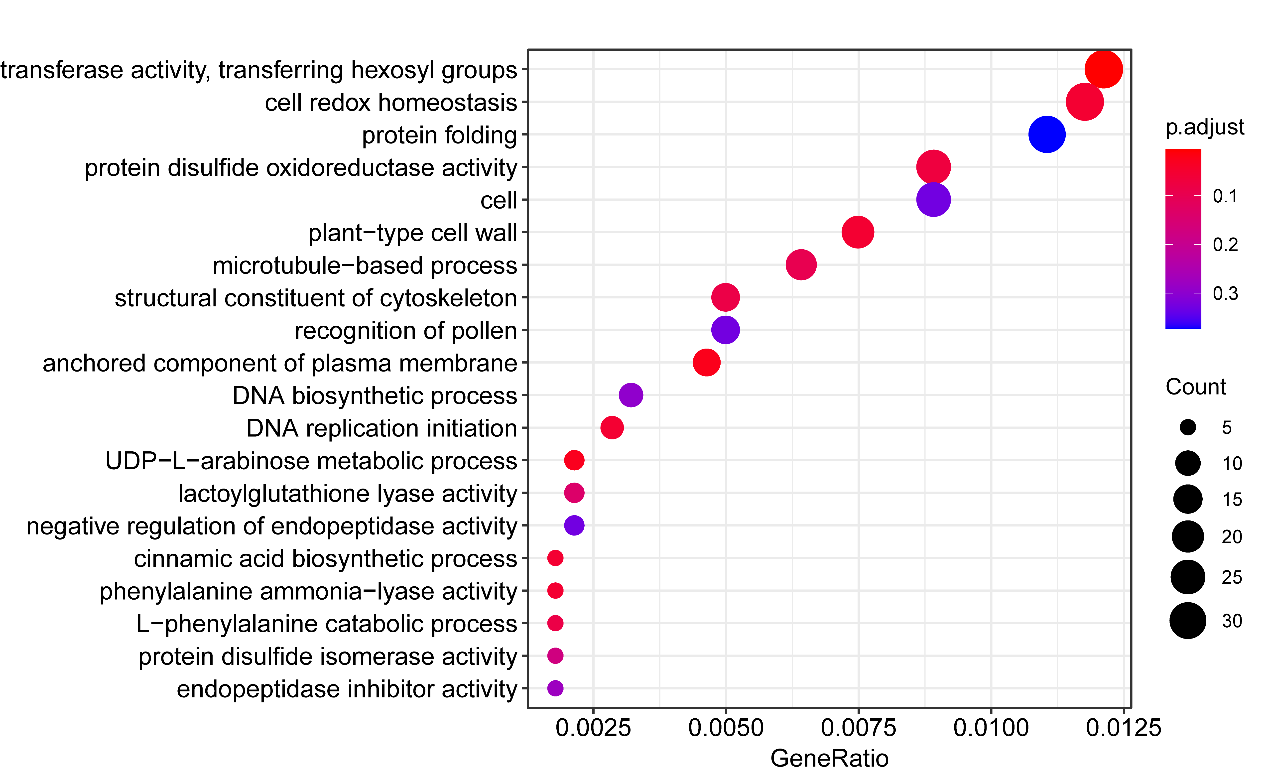


**Figure S21. GO Enrichment result of DMR-associated DEGs.** Circle sizes indicated gene numbers overlapped with the GO item group. Color indicates the adjust *P*-values for hypergeometric test and FDR adjustments.


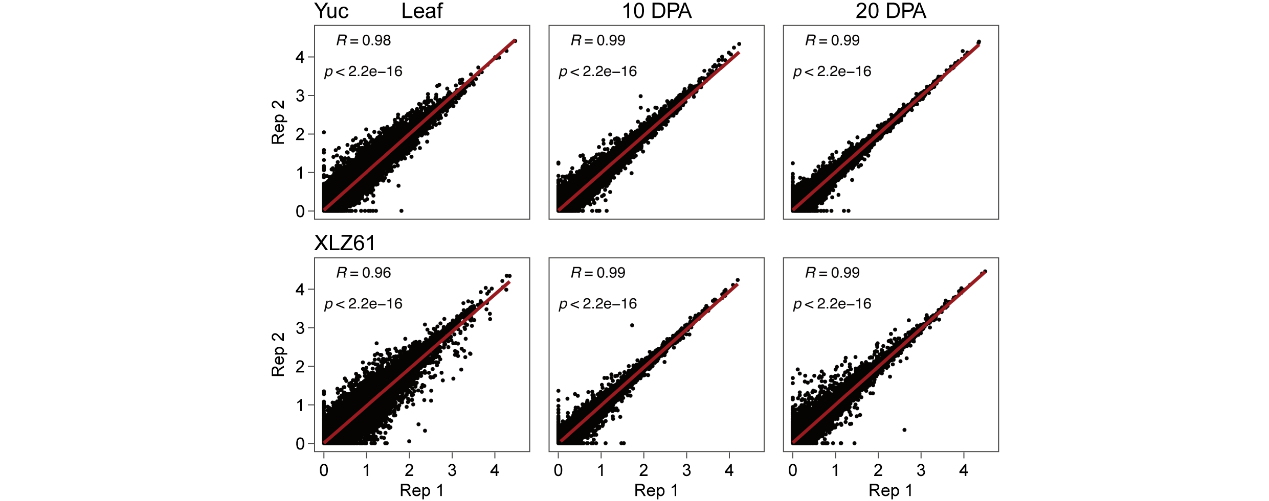


**Figure S22 Pearson correlation analysis between two replicates.** R indicates the Pearson correlation coefficient

**
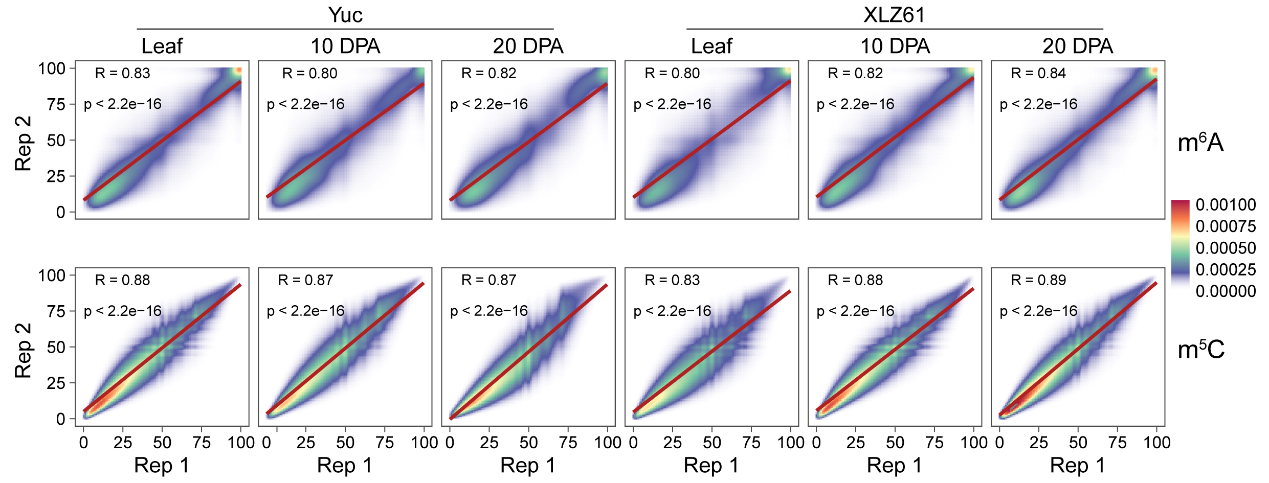
**

Figure S23 Pearson correlation analysis of RNA methylation level between two replicates.


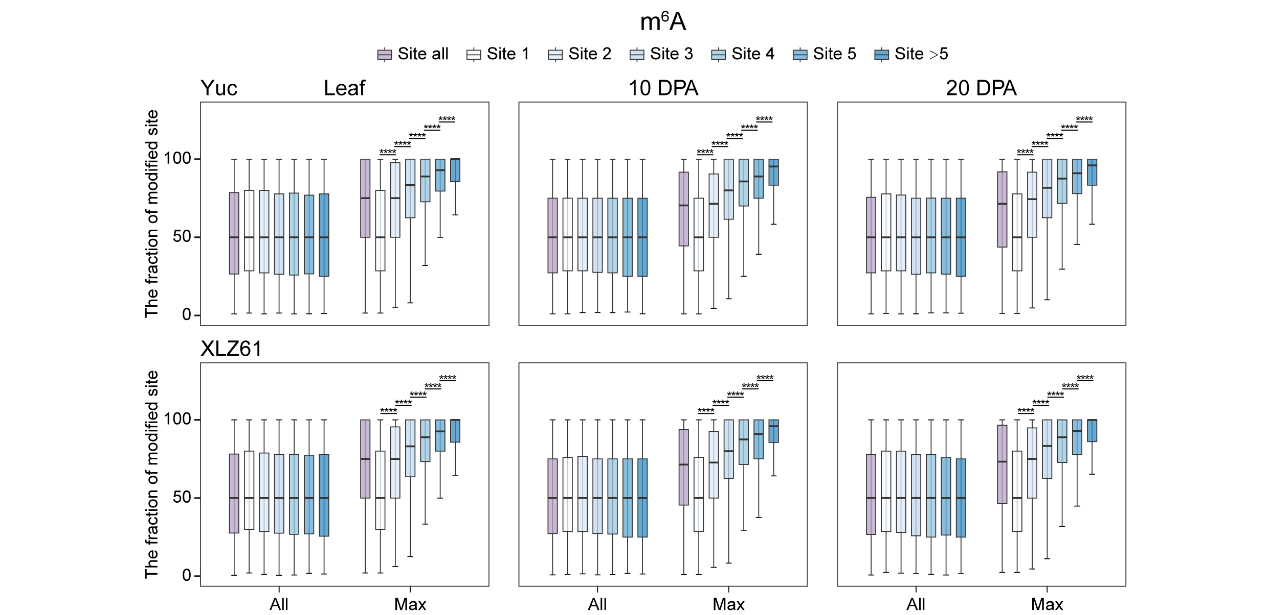


**Figure S24 Comparing the the max fraction of the transcripts with different m^6^A modified sites.** The fraction of m^6^A-modified sites in each transcript was calculated, and the maximum fraction in each transcript was counted. The significance was determined by t-test. **** indicated the *p* <0.0001.


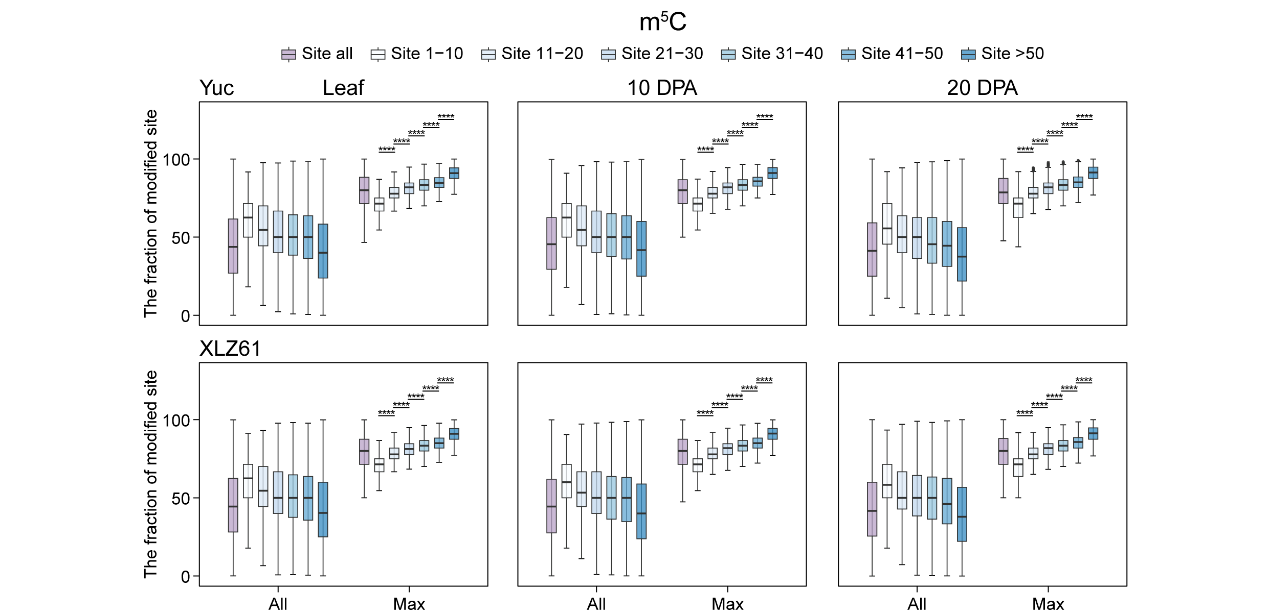


**Figure S25 Comparing the the max fraction of the transcripts with different m^5^C modified sites.** The fraction of m^5^C-modified sites in each transcript was calculated, and the maximum fraction in each transcript was counted. The significance was determined by t-test. **** indicated the *p* <0.0001.


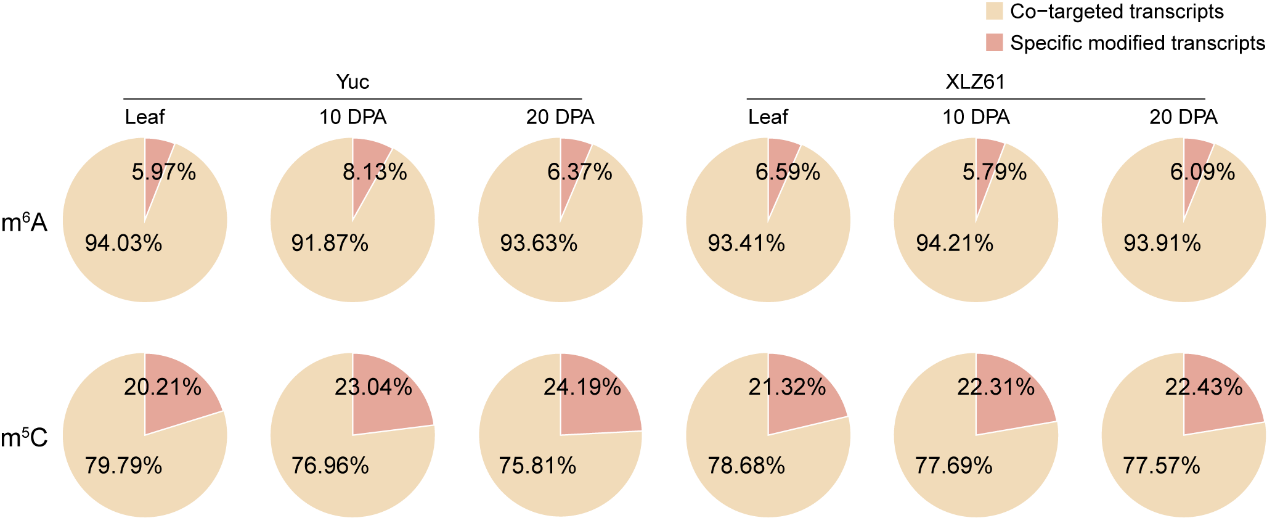


Figure S26 Ratio of transcripts with co-targeted of m^6^A and m^5^C methylation in different tissues.

**
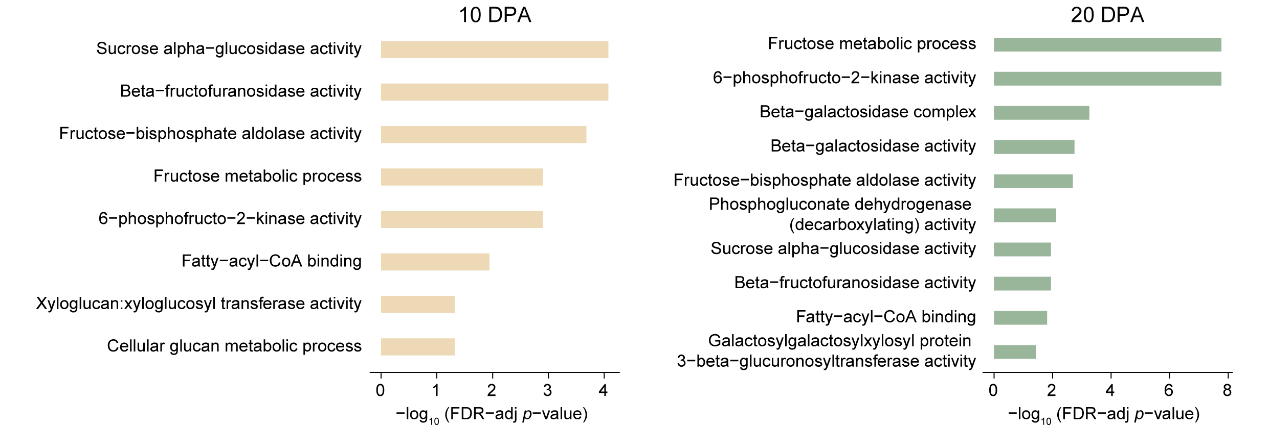
**

Figure S27 GO enrichment of transripts with DMLs


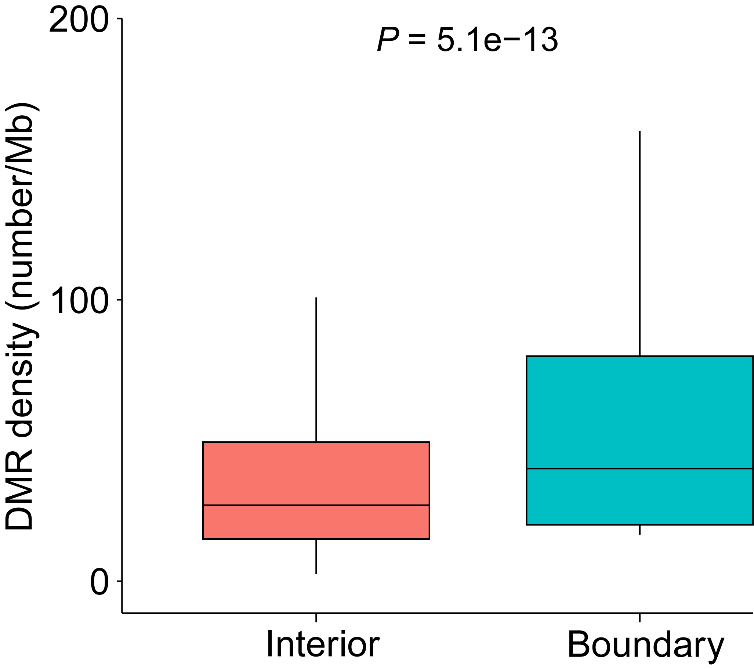


**Figure S28. Comparative analysis of the density of DMR with interior and boundary of differential TADs.** The significance was determined by t-test.

**
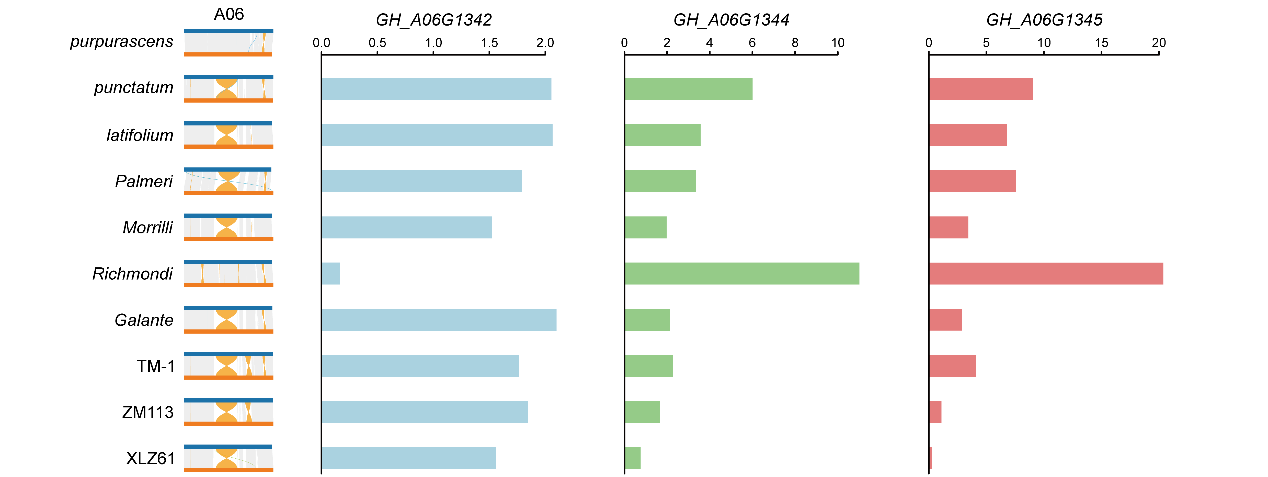
**

**Figure S29 The distribution of the largest inversion and its impact on the expression of *GH_A06G1342*, *GH_A06G1344* and *GH_A06G1345*.**


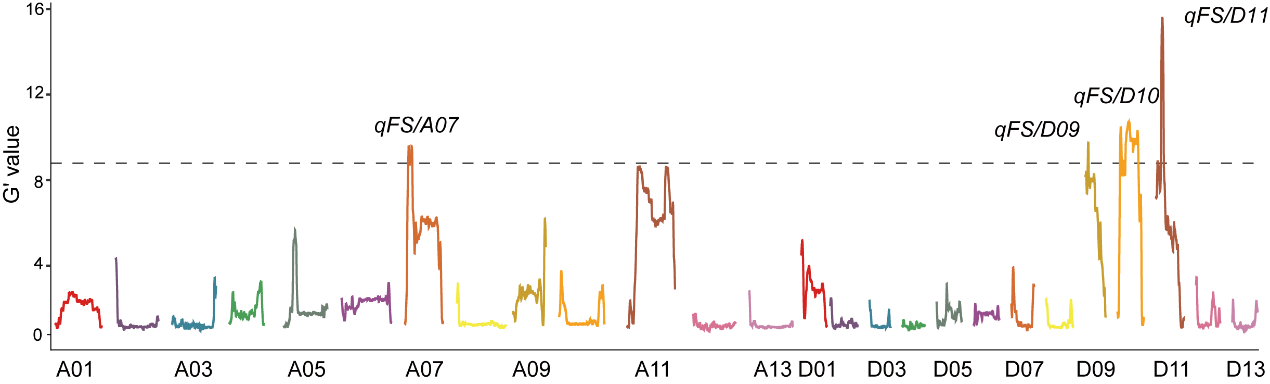


**Figure S30 QTL mapping for fiber strength using BSA analysis.** Genome-wide distribution of G’ values calculated from BSA-seq data. The horizontal red line represents the significance threshold (*P* < 0.001).


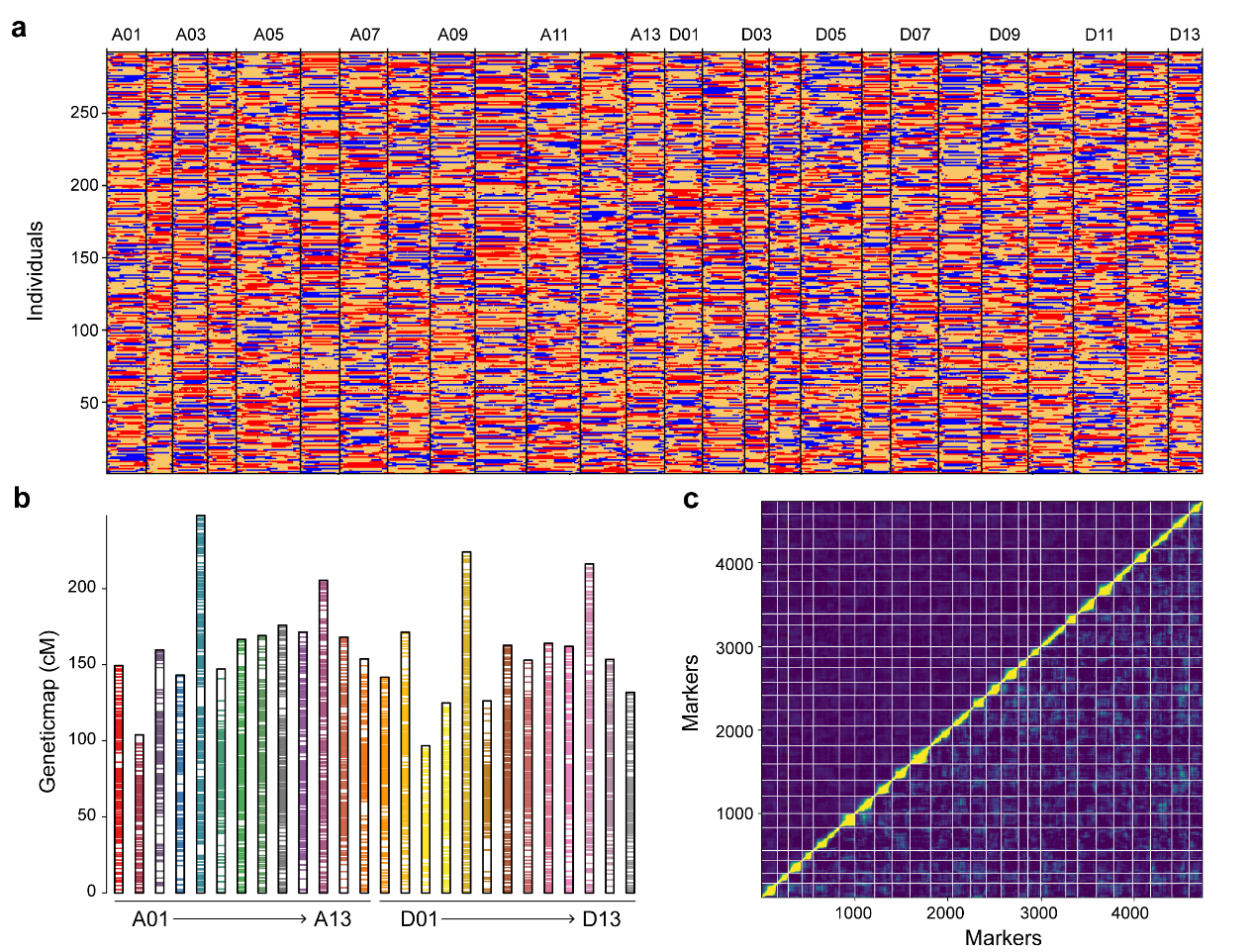


**Figure S31** **Construction and validation of (XLZ61 × Yuc)F_2_ geneticmap**. **a** Recombination SNP maps of 300 (XLZ61 × Yuc)F_2_ individuals. Red and blue present as XLZ61 and Yuc, respectively, and yellow: heterozygous genotype. **b** Genetic maps for the (XLZ61 × Yuc)F_2_ populations. The y-axis represents the genetic distance along each chromosome. **c** Genetic map quality as indicated by recombination fractions of all markers.


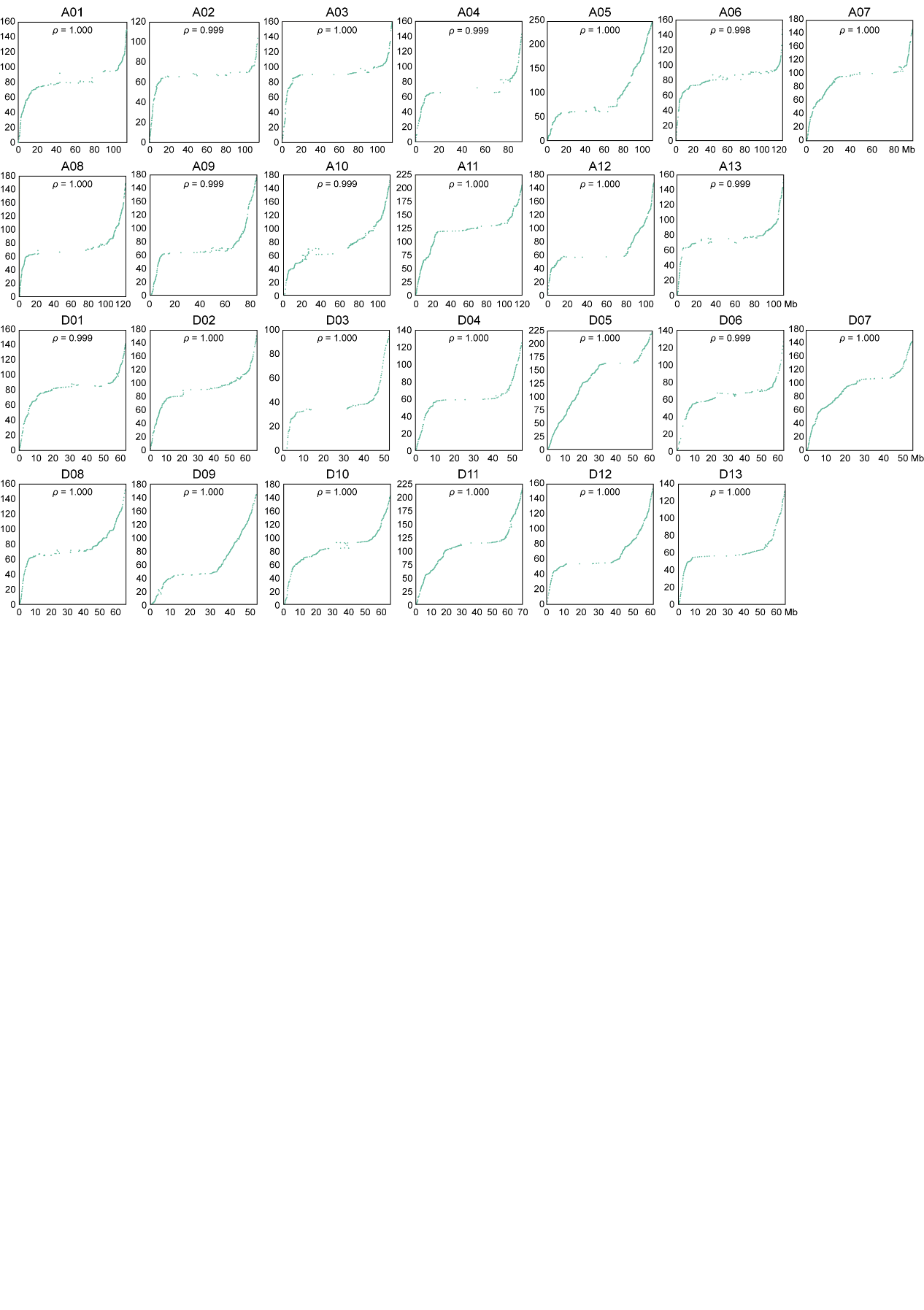


Figure S32 Comparisons of the TM-1 genome with (XLZ61 × Yuc)F_2_ genetic map


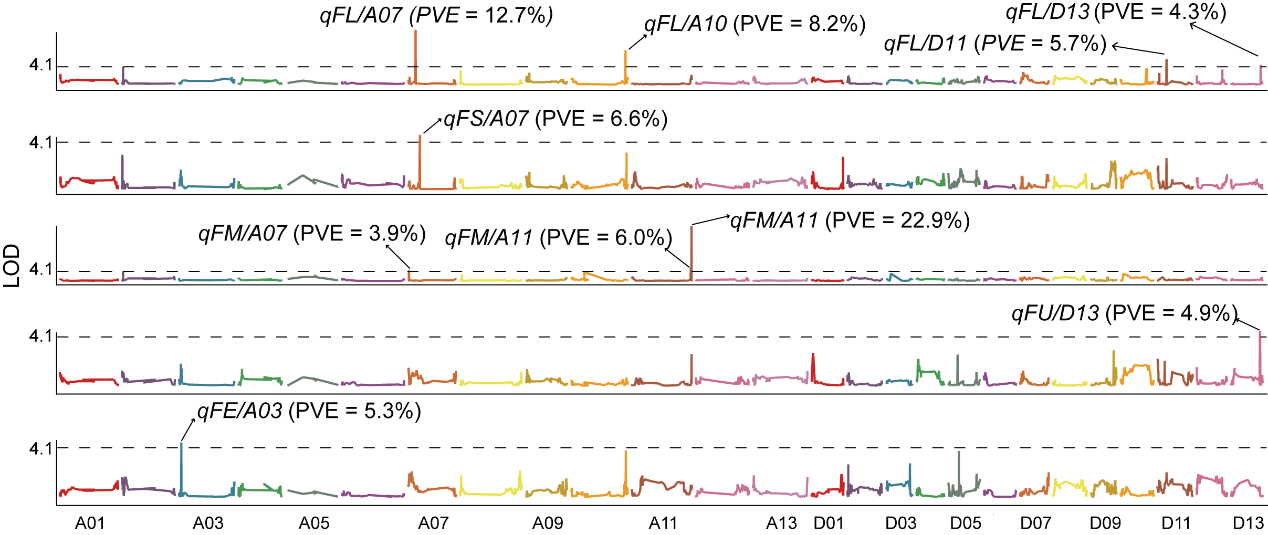


Figure S33 Identification of QTLs related to fiber quality by geneticmap-based QTL mapping.


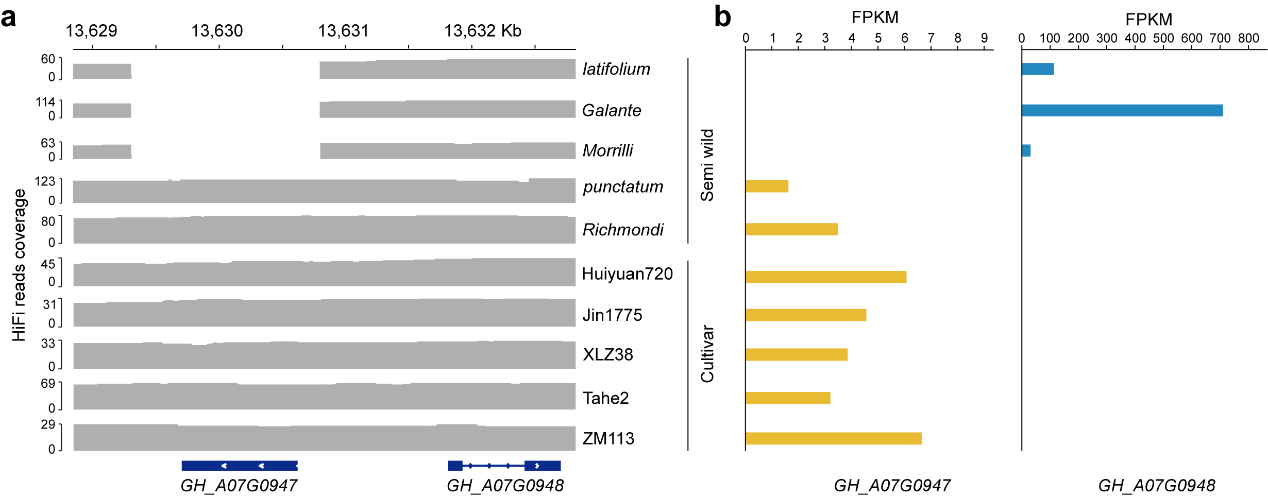


**Figure S34 The impact of a 1,706 bp insertion on the expression of *GH_A07G0947* and *GH_A07G0948* in five semi wild cottons and five cultivated cottons. a** Coverage of HiFi reads in the region contained *GH_A07G0947* and *GH_A07G0948*. **b** The expression of *GH_A07G0947* and *GH_A07G0948* in five semi wild cottons and five cultivated cottons.

***
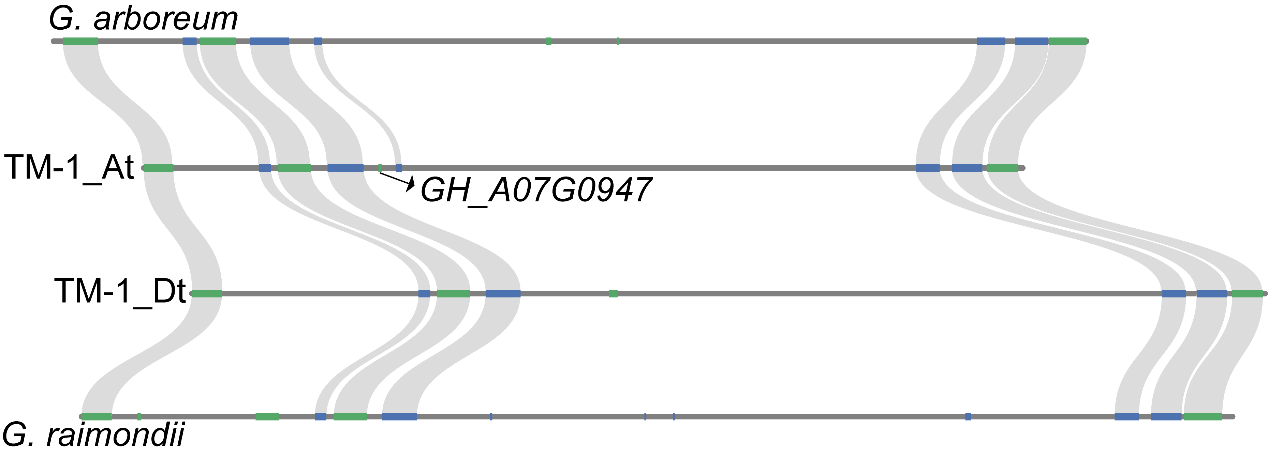
***

**Figure S35 Micro-synteny plot of gene *GH_A07G0947* among ancestral diploid cotton and allotetraploid cotton**


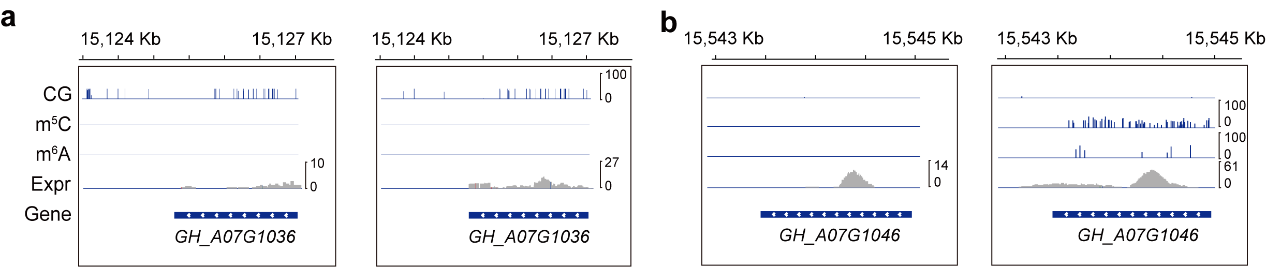


**Figure S36 Examples of DEG primarily caused by DNA (a) and RNA (b) methylation differences.** The first layer represents the distribution of CG methylation, the second layer represents the distribution of m^5^C methylation, the third layer represents the distribution of m^6^A methylation, the fourth layer represents expression levels, and the fifth layer represents gene location.
